# Fourteen new species of the spider genus *Thaiderces* from Southeast Asia (Araneae, Psilodercidae)

**DOI:** 10.3897/zookeys.869.35546

**Published:** 2019-08-05

**Authors:** Wan-Jin Chang, Shuqiang Li

**Affiliations:** 1 Institute of Zoology, Chinese Academy of Sciences, Beijing 100101, China Institute of Zoology, Chinese Academy of Sciences Beijing China

**Keywords:** endemic, Indonesia, Myanmar, new combination, Ochyroceratidae, Sumatra, Thailand, tropical

## Abstract

Fourteen new species of the genus *Thaiderces* F.Y. Li & S.Q. Li, 2017 from Indonesia, Myanmar, and Thailand are described: *T. shuzi* Li & Chang, **sp. nov.** (♂♀), *T. peterjaegeri* Li & Chang, **sp. nov.** (♂), *T. ganlan* Li & Chang, **sp. nov.** (♂♀), *T. ngalauindahensis* Li & Chang, **sp. nov.** (♂), *T. yangcong* Li & Chang, **sp. nov.** (♂♀), *T. zuichun* Li & Chang, **sp. nov.** (♀), *T. miantiao* Li & Chang, **sp. nov.** (♀), *T. jiazi* Li & Chang, **sp. nov.** (♀), *T. tuoyuan* Li & Chang, **sp. nov.** (♂♀), *T. fengniao* Li & Chang, **sp. nov.** (♂♀), *T. haima* Li & Chang, **sp. nov.** (♂♀), *T. chujiao* Li & Chang, **sp. nov.** (♀), *T. thamphadaengensis* Li & Chang, **sp. nov.** (♂♀), and *T. thamprikensis* Li & Chang, **sp. nov.** (♂♀). In addition, two species of *Psiloderces* Simon, 1892 are transferred to *Thaiderces*: *Thaiderces rimbu* (Deeleman-Reinhold, 1995) **comb. nov.** (♂♀) and *Thaiderces djojosudharmoi* (Deeleman-Reinhold, 1995) **comb. nov**. (♂♀).

## Introduction

The spider family Psilodercidae Machado, 1951 was recently elevated from a subfamily of Ochyroceratidae Fage, 1912 to family rank (Wunderlich 2004, 2008). Psilodercidae currently includes 127 species belonging to eleven genera (Li and Quan 2017; WSC 2019). All species are restricted to tropical Asia. More than half the genera were described only recently: *Flexicrurum* Y.F. Tong & S.Q. Li, 2007, *Luzonacera* F.Y. Li & S.Q. Li, 2017, *Priscaleclercera* Wunderlich, 2017, *Qiongocera* F.Y. Li & S.Q. Li, 2017, *Relictocera* F.Y. Li & S.Q. Li, 2017, *Sinoderces* F.Y. Li & S.Q. Li, 2017, and *Thaiderces* F.Y. Li & S.Q. Li, 2017 (Liu et al. 2017).

Prior to this study, only two species of *Thaiderces* were known: *T. jian* Li & Li, 2017 and *T. vulgaris* (Deeleman-Reinhold, 1995) (WSC 2019). Both species are endemic to Thailand. While studying new material collected in Southeast Asia, we found fourteen new species of *Thaiderces* from Myanmar, Thailand, and Sumatra Island of Indonesia. The goal of this paper is to provide detailed descriptions of these new species.

## Materials and methods

Types are deposited in the Institute of Zoology, Chinese Academy of Sciences (**IZCAS**) in Beijing, except *Thaiderces peterjaegeri* sp. nov. which is lodged in the Senckenberg Research Institute in Frankfurt, Germany (**SMF**). All specimens collected were preserved and observed in a 95% ethanol solution. The specimens were measured and examined under a Leica M205 C stereomicroscope, and further morphological details were observed using an Olympus BX41 compound microscope. The left male palp was dissected for further examination. The carapace measurements exclude the clypeus. The endogyne and the male palp were dissected and immersed in lactic acid for digestion. An Olympus C7070 wide zoom digital camera (7.1 megapixels) mounted on an Olympus SZX12 stereomicroscope was used to take photos at different focal planes. The photos were assembled with the image stacking software Helicon Focus 6.7.1. to generate high quality photos before further revision with Adobe Photoshop CC 2014. Leg measurements are given as total length (femur, patella, tibia, metatarsus, and tarsus). Leg segments were measured from their retrolateral side. All measurements are given in millimetres (mm). All terminology follows Li et al. (2014).

## Taxonomy

### Family Psilodercidae Machado, 1951

#### Genus Thaiderces

Taxon classificationAnimaliaAraneaePsilodercidae

F.Y. Li & S.Q. Li, 2017

7ACF293343F950A797BA7F888C08FB23

##### Type species.

**Figure 1.**
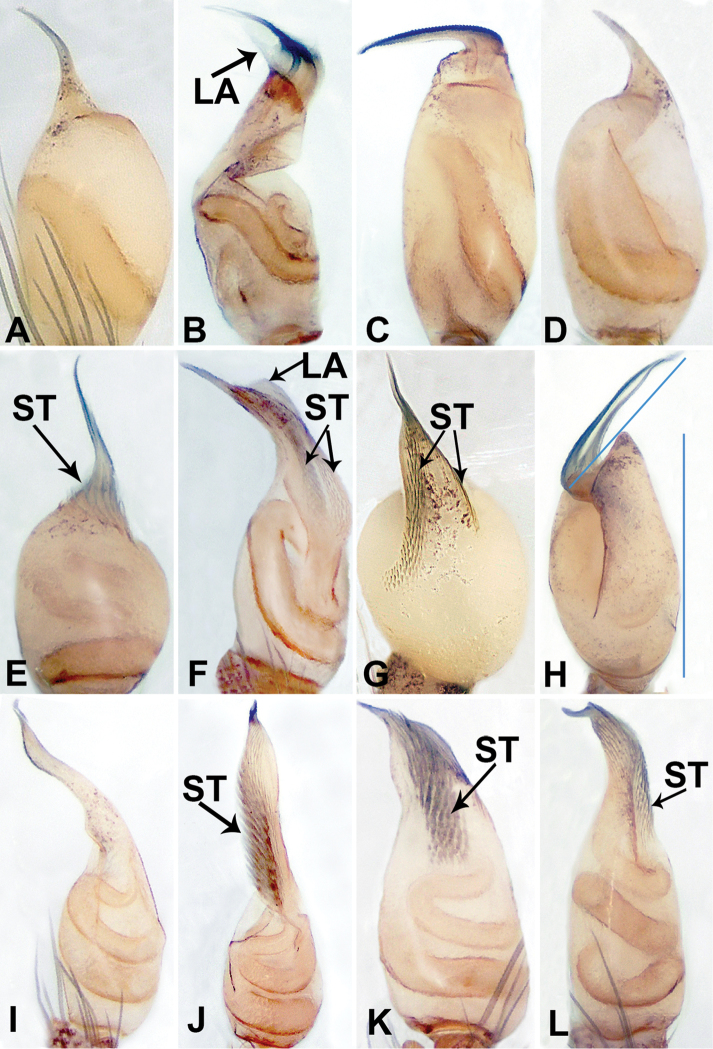
Prolateral view of left bulb in *Thaiderces* species **A***T. jian***B***T. ngalauindahensis* sp. nov. **C***T. peterjaegeri* sp. nov. **D***T. tuoyuan* sp. nov. **E***T. yangcong* sp. nov. **F***T. fengniao* sp. nov. Ventral view of the bulb in *Thaiderces* species **G***T. vulgaris***H***T. ganlan* sp. nov. **I***T. thamphadaengensis* sp. nov. **J***T. shuzi* sp. nov. **K***T. thamphrikensis* sp. nov. **L***T. haima* sp. nov. Abbreviations: LA, laminal apophysis, ST, stubble. **A, G** Modified from Liu et al. (2017).

**Figure 2.**
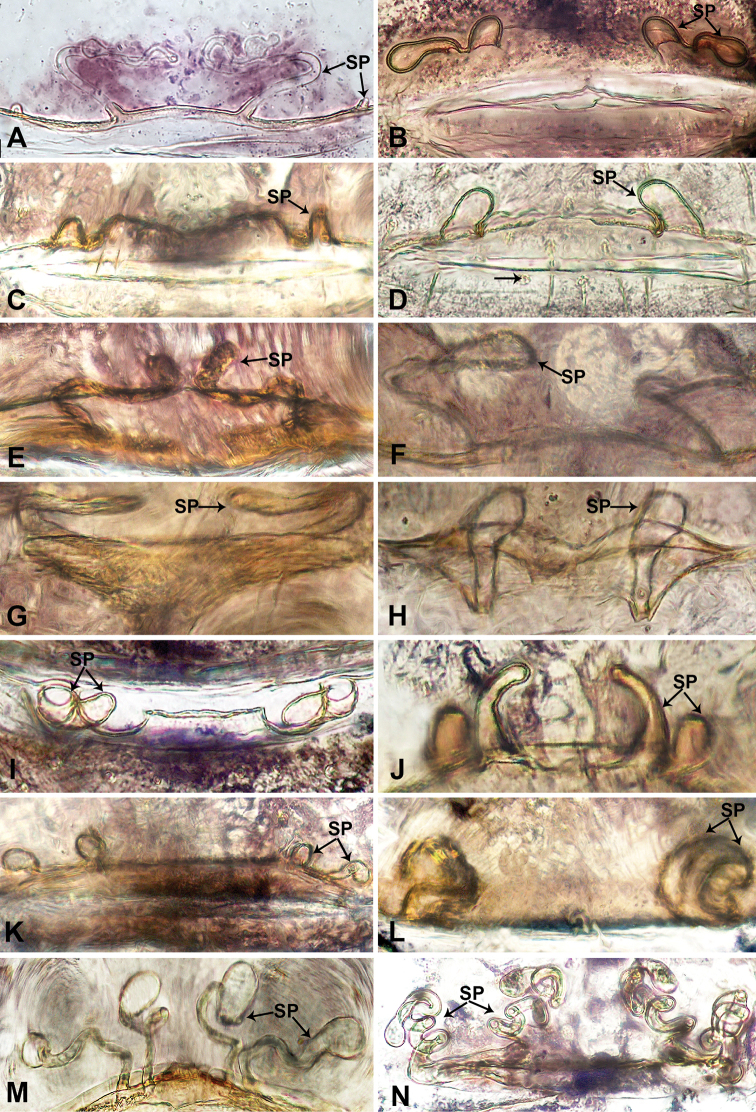
Endogyne of *Thaiderces* species **A***T. jian***B***T. vulgaris***C***T. thamprikensis* sp. nov. **D***T. fengniao* sp. nov. **E***T. ganlan* sp. nov. **F***T. tuoyuan* sp. nov. **G***T. jiazi* sp. nov. **H***T. zuichun* sp. nov. **I***T. haima* sp.nov. **J***T. chujiao* sp. nov. **K***T. thamphadaengensis* sp. nov. **L***T. yangcong* sp. nov. **M***T. shuzi* sp. nov. **N***T. miantiao* sp. nov. Abbreviation: SP, spermathecae. **A, B** Modified from Liu et al. (2017).

*Thaiderces jian* from Thailand, details and figures of the type species as in figs 1A, 2A, and Liu et al. (2017: figs 11–12).

##### Emended diagnosis.

*Thaiderces* resembles *Sinoderces* by the absence of an apical protrusion on the cymbium, cheliceral lamina with 3 triangular extensions, shallow fovea, and clypeus and labium slanting, but it can be differentiated by the following combination of characters: 1) presence of embolic stalk or embolic ‘stubble’ (vs. absence of embolic stalk or ‘stubble’); 2) absence of setae on ocular region (vs. presence of numerous setae); 3) embolus shorter than bulb (vs. embolus longer than bulb); 4) absence of conductor (vs. presence); and 5) 3 retromarginal cheliceral teeth (vs. one retromarginal tooth).

##### Composition.

*Thaiderces jian* (♂♀) (the type species), *T. vulgaris* (Deeleman-Reinhold, 1995) (♂♀), *T. shuzi* sp. nov. (♂♀), *T. peterjaegeri* sp. nov. (♂), *T. ganlan* sp. nov. (♂♀), *T. ngalauindahensis* sp. nov. (♂), *T. yangcong* sp. nov. (♂♀), *T. tuoyuan* sp. nov. (♂♀), *T. fengniao* sp. nov. (♂♀), *T. haima* sp. nov. (♂♀), *T. thamphadaengensis* sp. nov. (♂♀), *T. thamphrikensis* sp. nov. (♂♀), *T. jiazi* sp. nov. (♀), *T. zuichun* sp. nov. (♀), *T. chujiao* sp. nov. (♀), and *T. miantiao* sp. nov. (♀).

##### Distribution.

Thailand, Myanmar, and Sumatra Island of Indonesia.

### Key to species of *Thaiderces*, males only

**Table:** 

1	Embolic stubble absent	**2**
–	Embolic stubble present	**7**
2	Embolus is shorter than bulb	**3**
–	Embolus and bulb almost equal in length (Fig. 1H)	***T. ganlan* sp. nov.**
3	Embolus with long stalk	**4**
–	Embolus without stalk	**5**
4	Bulb with lamina, embolic stalk with distinct inclination of 30° (Fig. 1B)	***T. ngalauindahensis* sp. nov.**
–	Bulb without lamina, embolic stalk flat and tapering (Fig. 1I)	***T. thamphadaengensis* sp. nov.**
5	Embolus thin, straight and elongated, perpendicular to the axis of the bulb (Fig. 1C)	***T. peterjaegeri* sp. nov.**
–	Embolus thick, curved and short, directed upward	**6**
6	Bulb obovate; embolus originating prolaterally (Fig. 1D)	***T. tuoyuan* sp. nov.**
–	Bulb oblong; embolus originating retrolaterally (Fig. 1A)	*** T. jian ***
7	Embolic stalk long	**8**
–	Embolic stalk absent (Fig. 1E)	***T. yangcong* sp. nov.**
8	Embolic stubble divided into 2 rows	**9**
–	Embolic stubble not divided	**10**
9	Bulb with lamina (Fig. 1F)	***T. fengniao* sp. nov.**
–	Bulb without lamina (Fig. 1G)	*** T. vulgaris ***
10	Embolic stubble serrated, covers the entire embolic stalk (Fig. 1J)	***T. shuzi* sp. nov.**
–	Embolic stubble not serrated, covers only distal end of embolic stalk	**11**
11	Embolic stubble aligned, almost as wide as bulb (Fig. 1K)	***T. thamphrikensis* sp. nov.**
–	Embolic stubble not aligned, 3 times thinner than bulb (Fig. 1L)	***T. haima* sp. nov.**

### Key to species of *Thaiderces*, females only

**Table:** 

1	One pair of spermathecae	**2**
–	Two pairs of spermathecae	**7**
2	Spermathecae tubular, without stalks	**3**
–	Spermathecae with stalks	**4**
3	Wavy ducts present medially, connected with tubular spermathecae laterally (Fig. 2C)	***T. thamprikensis* sp. nov.**
–	Wavy ducts lacking, tubular spermathecae bend towards each other (Fig. 2D)	***T. fengniao* sp. nov.**
4	Spermathecae with twisted stalks	**5**
–	Stalk of spermatheca not twisted or simply bent at a right angle	**6**
5	Spermathecae with globular heads 2 times wider than stalk (Fig. 2E)	***T. ganlan* sp. nov.**
–	Spermathecae with globular heads 3 times wider than stalk (Fig. 2F)	***T. tuoyuan* sp. nov.**
6	Spermathecae connected by a funnel-like base (Fig. 2G)	***T. jiazi* sp. nov.**
–	Spermathecae connected by a wavy horizontal duct (Fig. 2H)	***T. zuichun* sp. nov.**
7	Spermathecae without stalks, tubular	**8**
–	Spermathecae with stalks, twisted	**11**
8	Paired spermathecae touching	**9**
–	Paired spermathecae separated	.**10**
9	Lateral spermathecae similar to median spermathecae but embedded with ovoid duct structure (Fig. 2I)	***T. haima* sp. nov.**
–	Lateral spermathecae distinctly shorter and wider than median spermathecae (Fig. 2J)	***T. chujiao* sp. nov.**
10	Lateral spermathecae with stalks (Fig. 2K)	***T. thamphadaengensis* sp. nov.**
–	Lateral spermathecae 2 times longer than median pair and directed horizontally (Fig. 2B)	*** T. vulgaris ***
11	Lateral and median spermathecae of similar shape	**12**
–	Lateral spermathecae short and tubular, median spermathecae long and twisted (Fig. 2A)	*** T. jian ***
12	Spermathecae with globose heads	**13**
–	Spermathecae without globose heads (Fig. 2L)	***T. yangcong* sp. nov.**
13	Heads of spermathecae 3 times wider than stalk (Fig. 2M)	***T. shuzi* sp. nov.**
–	Heads of spermathecae almost equally as wide as stalk (Fig. 2N)	***T. miantiao* sp. nov.**

#### Thaiderces shuzi

Taxon classificationAnimaliaAraneaePsilodercidae

Li & Chang sp. nov.

7646DCAA92EE573FBEA0FE113C398DD1

http://zoobank.org/D3DB4169-236B-4C6E-85ED-98C160CED4F3

**Figure 3.**
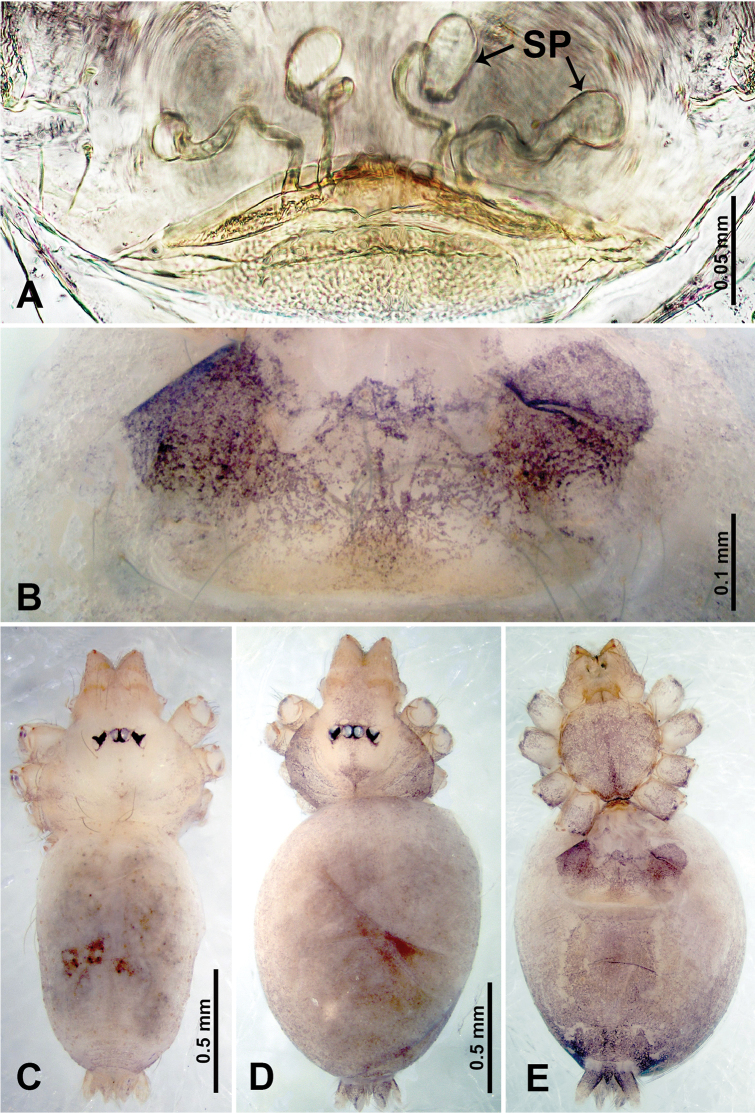
*Thaiderces shuzi* sp. nov., male holotype and female paratype **A** endogyne, dorsal view **B** female epigastric area, ventral view **C** male habitus, dorsal view **D** female habitus, dorsal view **E** female habitus, ventral view. Abbreviation: SP, spermatheca.

**Figure 4.**
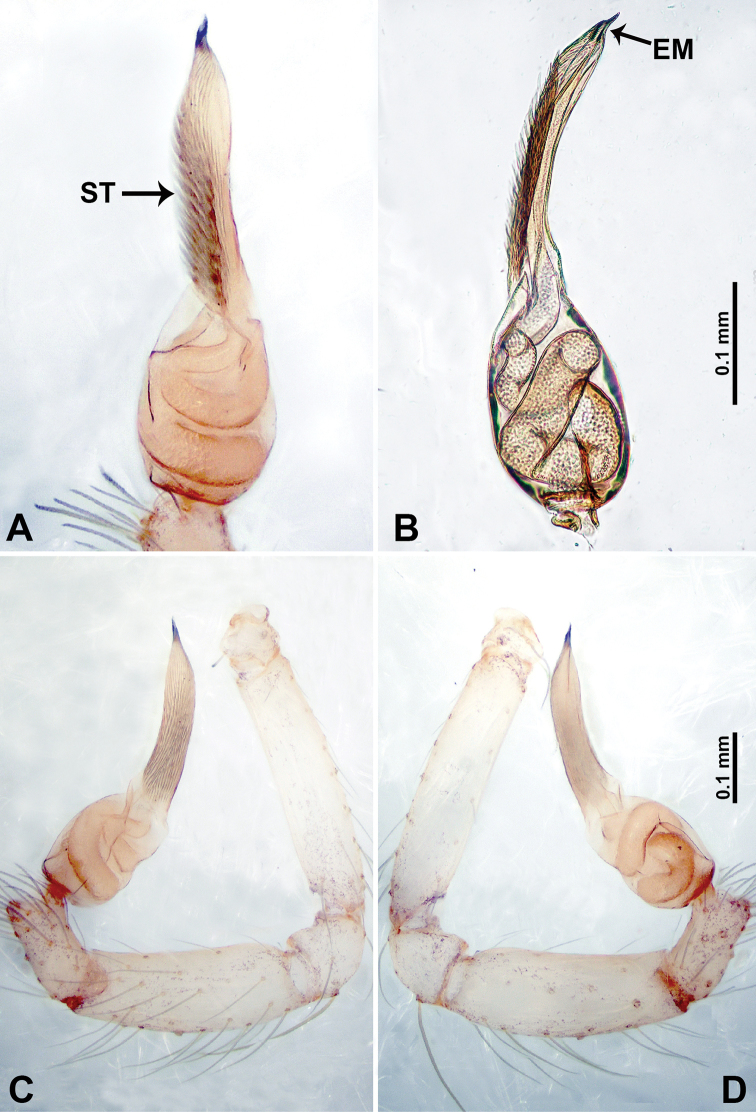
*Thaiderces shuzi* sp. nov., male holotype **A** palp, ventral view **B** palpal bulb, ventral view **C** palp, prolateral view **D** palp, retrolateral view. Abbreviations: EM, embolus, ST, stubble.

Figs 1J , 2M , 3 , 4 , 27C , 29

##### Types.

**Holotype**: ♂ (IZCAS), Thailand, Prachuap Kiri Khan Province, Hua Hin District, Nong Phiap Subdistrict, Dao Cave, 12°35.449'N, 99°43.692'E, 123 m, 30.X.2014, Zhao H., Li Y., and Chen Z. **Paratype**: 1♀ (IZCAS), same data as holotype.

##### Etymology.

The species name is a noun in apposition derived from the Chinese pinyin *shūz*ǐ (comb) and refers to the serrated stubble on the embolic stalk that resembles a comb.

##### Diagnosis.

Males of *T. shuzi* sp. nov. can be distinguished from all other species of the genus by the unique structure of the male bulb with a long embolic stalk bearing serrated embolic stubble (Fig. 4A) that resembles a comb (vs. absence of serrated embolic stubble in congeners); females can be differentiated from congeners by two pairs of stalked spermathecae with globose distal parts (Fig. 3A) (vs. one pair of spermathecae or spermathecae without stalk in congeners).

##### Description.

**Male** (holotype). Total length 1.78; carapace 0.78 long, 0.70 wide; abdomen 1.00 long, 0.70 wide. Carapace round and pale yellow (Fig. 3C). Chelicerae brown (Fig. 27C). Clypeus pale yellow. Endites pale yellow. Labium light brown. Sternum with purplish pattern. Abdomen elongated, dorsum with 3 distinct brown spots medially (Fig. 3C), anteroventrally with a pair of circular purplish patches, posterior part with a pattern ranging from light purple to dark purple. Legs uniformly brown; measurements: I 5.29 (1.40, 0.20, 1.56, 1.25, 0.88), II 7.36 (2.00, 0.20, 2.19, 1.88, 1.09), III 5.27 (1.56, 0.31, 1.40, 1.30, 0.70), IV 7.25 (2.00, 0.25, 2.20, 1.80, 1.00). Palp (Fig. 4A–D): femur slender, 5 times longer than patella; patella not swollen, tibia almost equal in length to femur, basally swollen (length/width = 2.30); cymbium pale, three times shorter than femur; bulb light yellow, spatulate, with a long embolic stalk bearing serrated stubble, embolic stalk almost equal in length to the bulb, 2/3 the width of the bulb; embolus straight and short apically (Fig. 4B).

**Female** (Paratype). General features and coloration similar to that of male (Fig. 3D–E). Measurements: total length 2.00; carapace 0.70 long, 0.70 wide; abdomen 1.30 long, 1.00 wide. Leg measurements: I‒II missing, III 4.54 (1.28, 0.25, 1.25, 1.13, 0.63), IV 4.51 (1.25, 0.25, 1.25, 1.13, 0.63). Endogyne: two pairs of stalked spermathecae bearing globose distal parts, lateral spermathecae directed horizontally, medial spermathecae pointed vertically, width of globose part ca. 3 times wider than stalk. (Fig. 3A).

##### Distribution.

Known only from the type locality (Fig. 29).

#### Thaiderces peterjaegeri

Taxon classificationAnimaliaAraneaePsilodercidae

Li & Chang sp. nov.

281DEF7728F8517F98FB460DB48E9DCE

http://zoobank.org/6E65FBCD-43A5-4C8B-AB90-943E9F8EF1A1

**Figure 5.**
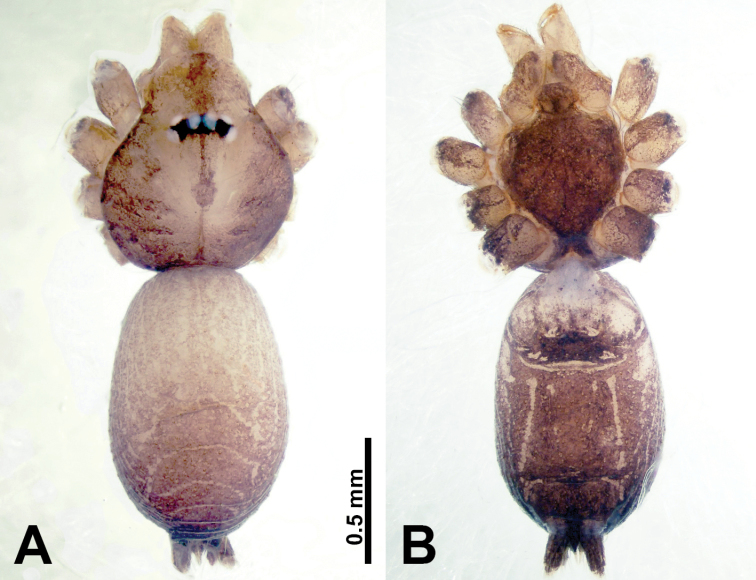
*Thaiderces peterjaegeri* sp. nov., male holotype **A** habitus, dorsal view **B** habitus, ventral view.

**Figure 6.**
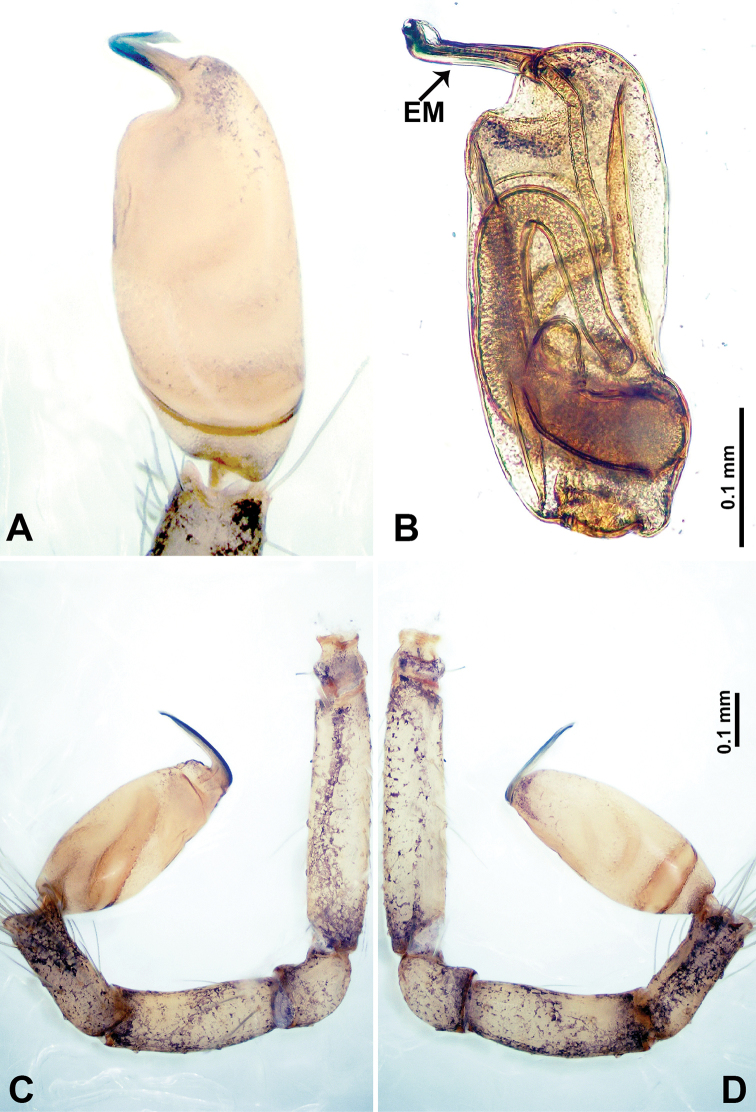
*Thaiderces peterjaegeri* sp. nov., male holotype **A** palp, ventral view **B** palpal bulb, ventral view **C** palp, prolateral view **D** palp, retrolateral view. Abbreviation: EM, embolus.

Figs 1C , 5 , 6 , 27E , 29

##### Types.

**Holotype**: ♂ (SMF), Myanmar, Chin State, Nat Ma Taung National Park, Road S of Nat Ma Taung Summit, Pristine Primary Forest, 21°10.125'N, 93°54.892'E, 2543 m, 16.V.2014, P. Jäger leg.

##### Etymology.

The species is named in honor of Peter Jäger (Frankfurt am Main, Germany), a prolific spider taxonomist.

##### Diagnosis.

Males of *T. peterjaegeri* sp. nov. resemble those of *T. ganlan* sp. nov. but can be distinguished by the narrow oblong shape of the bulb (Fig. 6C) (vs. elliptical in *T. ganlan* sp. nov. (Fig. 8C)), the embolus is half the length of the entire bulb (Fig. 6C) (vs. embolus almost equal in length to the entire bulb in *T. ganlan* sp. nov. (Fig. 8C)), and the embolus is thin and straight (Fig. 6C) (vs. embolus is thicker and curved in *T. ganlan* sp. nov. (Fig. 8B)).

##### Description.

**Male** (holotype). Total length 1.87; carapace 0.78 long, 0.75 wide; abdomen 1.09 long, 0.75 wide. Carapace round and brown, with 3 longitudinal brown bands, lateral bands 5 times wider than the middle band (Fig. 5A). Chelicerae brown (Fig. 27E). Clypeus light brown. Endites light brown. Labium dark brown. Sternum with dark brown pattern. Abdomen elongated, dorsum with gradual light to dark brown pattern extending from anterior to posterior (Fig. 5A), ventrum with gradual brown to dark brown pattern extending from anterior to posterior, anterior with elliptical dark brown patch medially, and a pair of lateral pale yellow patches, posterior part delimited with a pair of pale yellow straight lines (Fig. 5B). Legs uniformly brown. Measurements: I‒III missing, IV 3.80 (1.00, 0.20, 1.00, 1.00, 0.60). Palp with scattered purplish spots (Fig. 6C–D): femur four times longer than patella; patella not swollen, tibia 2/3 the length of femur; cymbium dark with concentrated purplish spots, half the length of femur; bulb light yellow, narrow, and oblong, length/width ratio = 1.90; embolus thin and straight, arises distally, half the length of the entire bulb (Fig. 6).

**Female.** Unknown.

##### Distribution.

Known only from the type locality (Fig. 29).

#### Thaiderces ganlan

Taxon classificationAnimaliaAraneaePsilodercidae

Li & Chang sp. nov.

61C0867A1057593EBFA14E5D6A7E39EB

http://zoobank.org/177CD9B3-65CA-4E72-B5FB-A253B6A0C7A0

**Figure 7.**
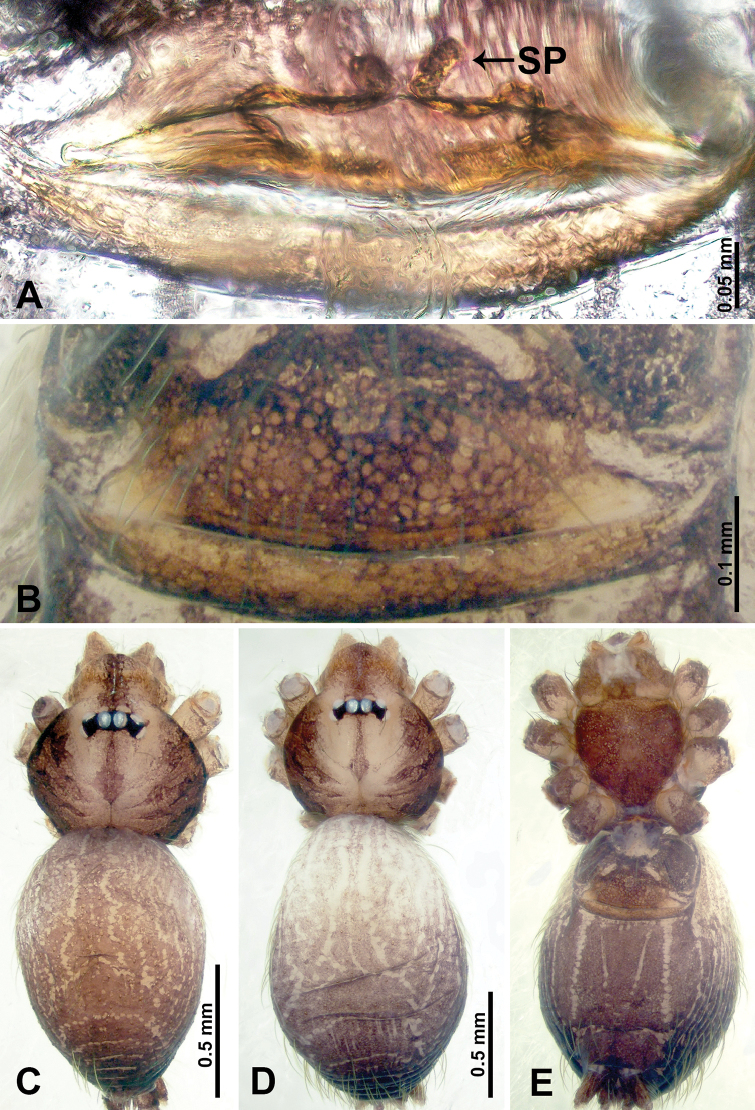
*Thaiderces ganlan* sp. nov., male holotype and female paratype **A** endogyne, dorsal view **B** female epigastric area, ventral view **C** male habitus, dorsal view **D** female habitus, dorsal view **E** female habitus, ventral view. Abbreviation: SP, spermatheca.

**Figure 8.**
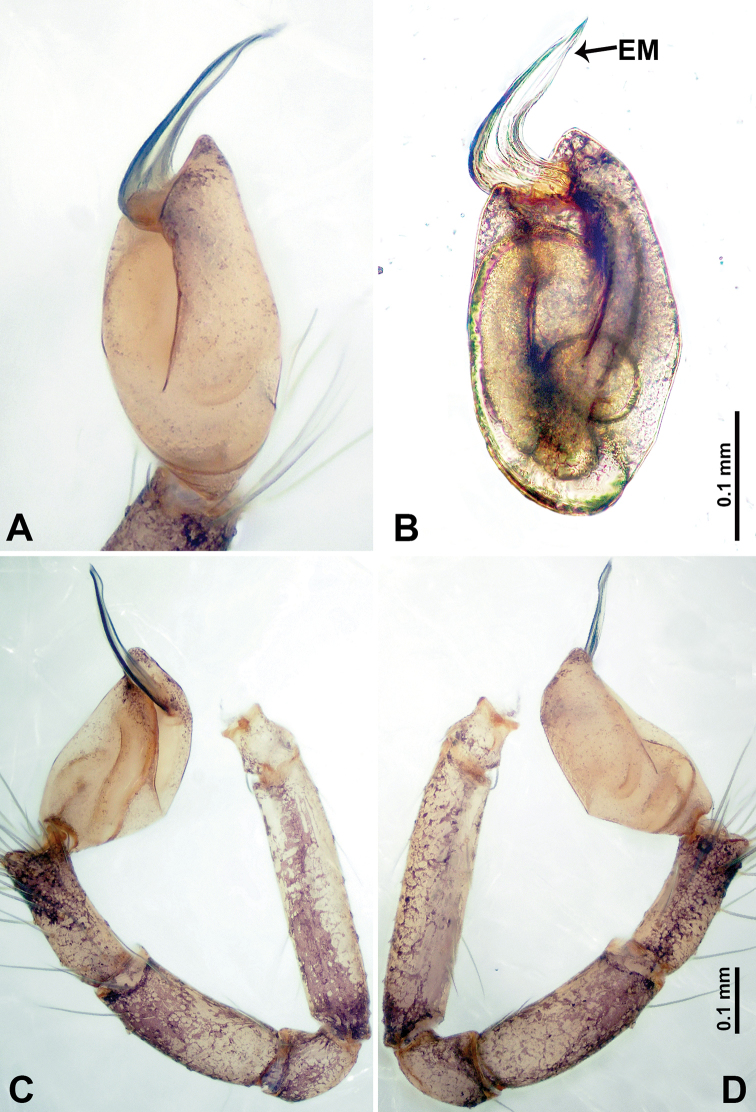
*Thaiderces ganlan* sp. nov., male holotype **A** palp, ventral view **B** palpal bulb, ventral view **C** palp, prolateral view **D** palp, retrolateral view. Abbreviation: EM, embolus.

Figs 1H , 2E , 7 , 8 , 28E , 29

##### Types.

**Holotype**: ♂ (IZCAS), Myanmar, Chin State, roadside between Kampellet to Nat Ma Taung National Park, 21°13.436'N, 93°58.819'E, 2402 m, 1.V.2017, Wu J. and Chen Z. **Paratype**: 1♀ (IZCAS), same data as holotype.

##### Etymology.

The species name is a noun in apposition derived from the Chinese pinyin *gănlăn* (olive) and refers to the structure of the bulb that resembles an olive or a rugby ball (Fig. 8A).

##### Diagnosis.

Diagnostic features of males are discussed under *T. peterjaegeri* sp. nov. Females of *T. ganlan* sp. nov. can be distinguished by a pair of twisted ribbon-liked spermathecae, with globular distal ends two times wider than stalk (vs. spermathecae with globular heads three times wider than stalk in *T. tuoyuan* sp. nov., Fig. 6A).

##### Description.

**Male** (holotype). Total length 1.63; carapace 0.63 long, 0.63 wide; abdomen 1.00 long, 0.75 wide. Carapace round and brown with 3 longitudinal brown bands, lateral bands three times wider than the median band (Fig. 7C). Chelicerae brown (Fig. 28E). Clypeus dark brown medially and light brown laterally. Endites light brown. Labium dark brown. Sternum with dark brown pattern. Abdomen brown and elongated, dorsum with complex yellow dotted patterns, anteroventrally brown with pair of dark brown circular patches followed by a light brown elliptical patch, posterior part with a pair of yellow dotted lines laterally and a V-shaped mark medially. Legs uniformly brown; measurements: I 5.23 (1.40, 0.20, 1.60, 1.25, 0.78), II 4.99 (1.09, 0.15, 1.41, 1.25, 1.09), III 3.54 (0.94, 0.25, 0.90, 0.90, 0.55), IV 4.80 (1.20, 0.20. 1.40, 1.25, 0.75). Palp with scattered purplish spots (Fig. 8C, D): femur three times longer than patella; patella not swollen, tibia 2/3 the length of femur; cymbium dark with concentrated purplish spots, half the length of femur; bulb light yellow and elliptical, with a distinct protrusion arising distally, adjacent to embolus; embolus thick and branched, arising distally, almost equal in length to the entire bulb (Fig. 8A).

**Female** (Paratype). General features and coloration similar to that of male (Fig. 7D, E). Measurements: total length 2.03; carapace 0.78 long, 0.75 wide; abdomen 1.25 long, 0.94 wide. Leg measurements: I 5.23 (1.28, 0.32, 1.60, 1.25, 0.78), II 4.84 (1.28, 0.32, 1.40, 1.09, 0.75), III missing, IV 5.61 (1.44, 0.32, 1.60, 1.50, 0.75). Endogyne with a pair of twisted ribbon-liked spermathecae with long stalks and globular heads, heads 2 times wider than stalks, stalks almost equal to the interdistance of stalk bases (Fig. 7A).

##### Distribution.

Known only from the type locality (Fig. 29).

#### Thaiderces ngalauindahensis

Taxon classificationAnimaliaAraneaePsilodercidae

Li & Chang sp. nov.

175094611CE05B3DAE38C39AE04261DE

http://zoobank.org/E59B7F10-44A0-442F-A698-54A4FA2130B3

**Figure 9.**
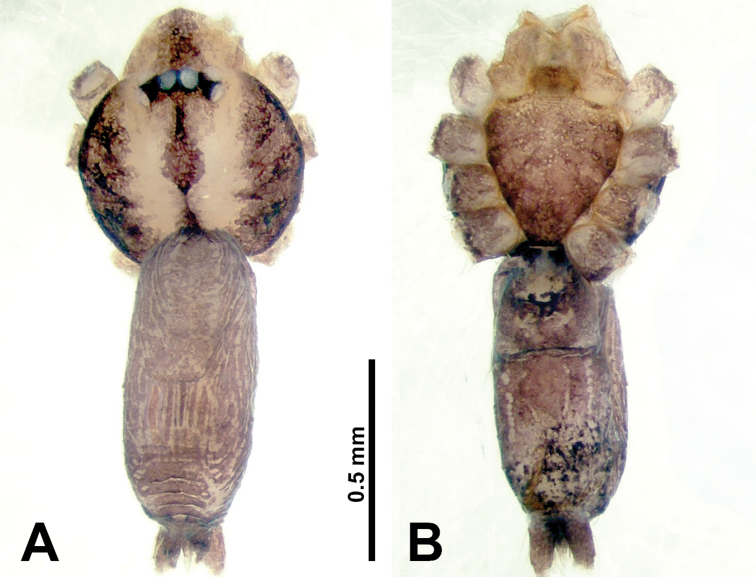
*Thaiderces ngalauindahensis* sp. nov., male holotype **A** habitus, dorsal view **B** habitus, ventral view.

**Figure 10.**
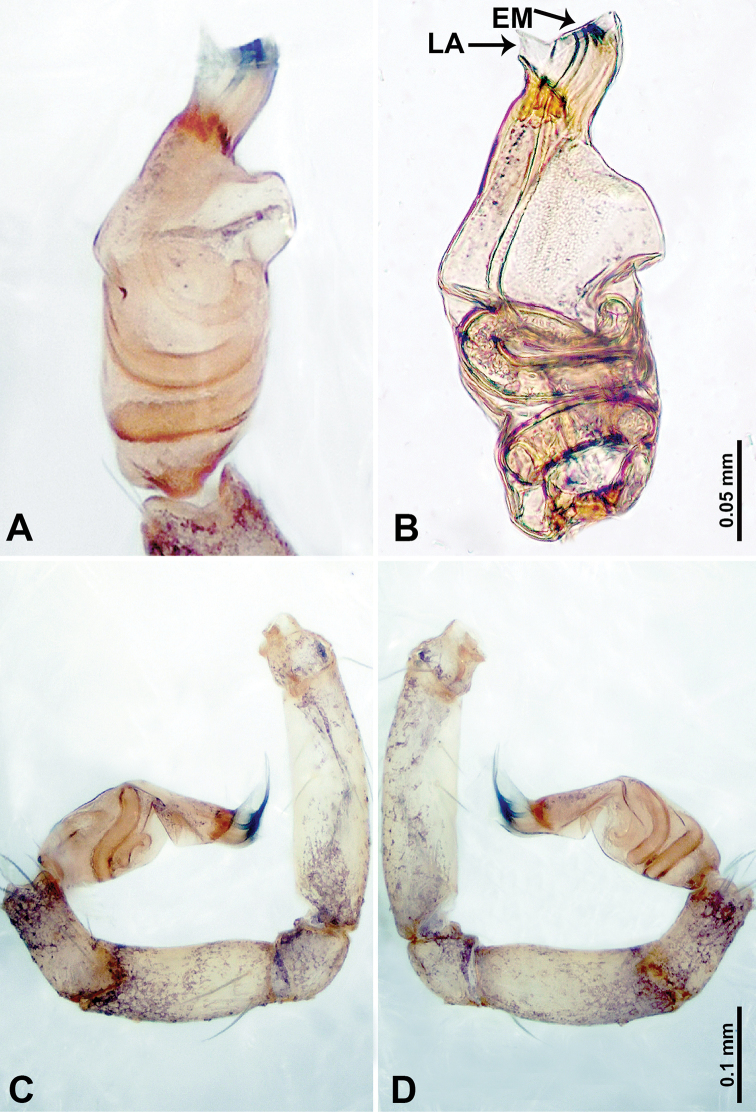
*Thaiderces ngalauindahensis* sp. nov., male holotype **A** palp, ventral view **B** palpal bulb, ventral view **C** palp, prolateral view **D** palp, retrolateral view. Abbreviations: EM, embolus, LA, lamina apophysis.

Figs 1B , 9 , 10 , 27F , 29

##### Types.

**Holotype**: ♂ (IZCAS), Indonesia, Sumatra, West Sumatra Province, Pavakumbuh, Ngalau Indah Cave, 00°15.296'S, 100°36.256'E, 626 m, 14.V.2014, Yao Z.

##### Etymology.

The species name is an adjective referring to the type locality.

##### Diagnosis.

*Thaiderces ngalauindahensis* sp. nov. is similar to *T. rimbu*, but males can be distinguished by lamina connected to the embolus (Fig. 10D) (vs. the absence of lamina connected to the embolus in *T. rimbu*) and the embolic stalk is slanted at a 30° incline (Fig. 10D) (vs. embolic stalk rather round and curved in *T. rimbu*).

##### Description.

**Male** (holotype). Total length 1.16; carapace 0.54 long, 0.55 wide; abdomen 0.62 long, 0.31 wide. Carapace round and brown, with three longitudinal dark brown bands, median band with distinct patch centrally, lateral bands four times wider than the middle band (Fig. 9A). Chelicerae brown (Fig. 27F). Clypeus dark brown medially and light brown laterally. Endites light brown. Labium dark brown. Sternum with dark brown pattern. Abdomen elongated, dorsum with dark brown complicated veined pattern (Fig. 9A), ventrum dark brown with indistinct pattern (Fig. 9B). Legs uniformly brown; measurements: I missing, II 3.35 (0.90, 0.20, 0.93, 0.78, 0.54), III missing, IV missing. Palp (Fig. 10C–D): femur four times longer than patella; patella not swollen, tibia almost equal in length to femur; cymbium with concentrated purplish spots, 1/2 the length of femur; bulb light brown, with 30° inclined embolic stalk, embolic stalk half the width of the bulb, lamina attached to embolus; embolus short and dark, arising distally (Fig. 10).

**Female.** Unknown.

##### Distribution.

Known only from the type locality (Fig. 29).

#### Thaiderces yangcong

Taxon classificationAnimaliaAraneaePsilodercidae

Li & Chang sp. nov.

3B9E4F809CE5555B983B19586AC9B8C5

http://zoobank.org/12BF7080-7804-4552-BF98-3ADE137B13C4

**Figure 11.**
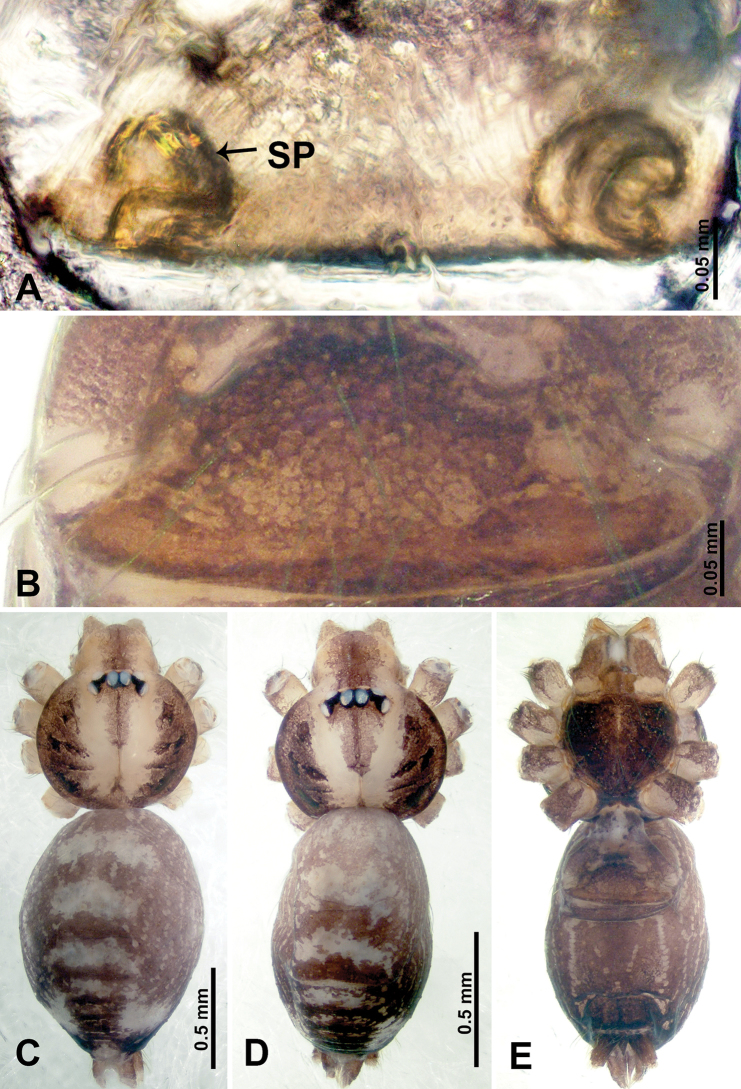
*Thaiderces yangcong* sp. nov., male holotype and female paratype **A** endogyne, dorsal view **B** female epigastric area, ventral view **C** male habitus, dorsal view **D** female habitus, dorsal view **E** female habitus, ventral view. Abbreviation: SP, spermatheca.

**Figure 12.**
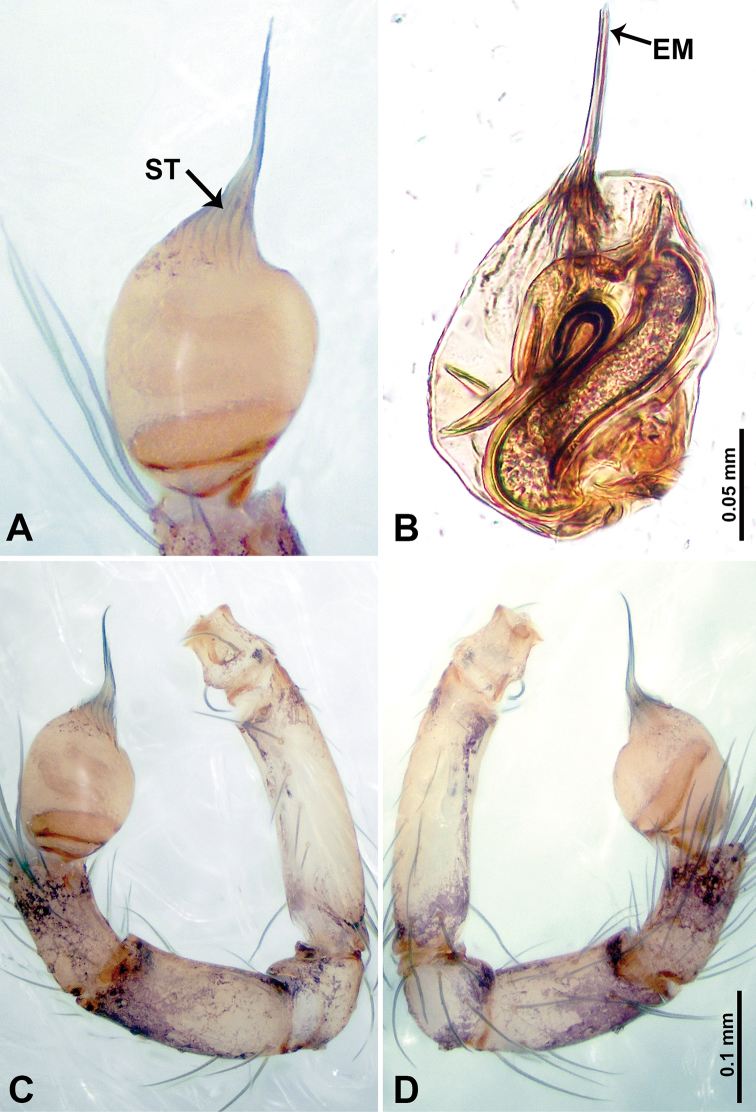
*Thaiderces yangcong* sp. nov., male holotype **A** palp, ventral view **B** palpal bulb, ventral view **C** palp, prolateral view **D** palp, retrolateral view. Abbreviations: EM, embolus, ST, stubble.

Figs 1E , 2L , 11 , 12 , 27B , 29

##### Types.

**Holotype**: ♂ (IZCAS), Indonesia, Sumatra, Jambi Province, Kerinci, Talang Cindang, near river, 02°04.834'S, 101°22.448'E, 1054 m, 23.V.2014, Yao Z. **Paratype**: 1♀ (IZCAS), same data as holotype.

##### Etymology.

The species name is a noun in apposition derived from the Chinese pinyin *yángcōng* (onion) and refers to the entire structure of the bulb which resembles an onion bulb.

##### Diagnosis.

*Thaiderces yangcong* sp. nov. is similar to *T. djojosudharmoi*, but males can be distinguished by the thin and long embolus (vs. a thick and short embolus in *T. djojosudharmoi*), a rather plump and rounded bulb (vs. a rather ovate bulb), and the presence of embolic stubble (vs. absence of embolic stubble); females can be distinguished by a pair of short, twisted, and rather distant wavy spermathecae (vs. two pairs of short petal-like spermathecae that are close together in *T. djojosudharmoi*).

##### Description.

**Male** (holotype). Total length 1.95; carapace 0.70 long, 0.75 wide; abdomen 1.25 long, 0.90 wide. Carapace round and pale brown, with three longitudinal dark brown bands, median band only half the length of carapace, lateral bands three times wider than the middle band (Fig. 11C). Chelicerae brown (Fig. 27B). Clypeus dark brown medially and light brown laterally. Endites pale yellow. Labium light brown. Sternum with dark brown pattern. Abdomen elongated, dorsum with brown stripes medially (Fig. 11C), anteroventrally brown with pair of dark brown circular patches followed by a light brown elliptical patch, posterior part with a pair of yellow dotted lines laterally and a V-shaped mark medially. Legs uniformly brown; measurements: I missing, II 5.15 (1.25, 0.16, 1.56, 1.40, 0.78), III 4.68 (1.25, 0.31, 1.25, 1.25, 0.62), IV 6.32 (1.56, 0.25, 1.87, 1.71, 0.93). Palp (Fig. 12A–D): femur four times longer than patella; patella not swollen, tibia 2/3 the length of femur; cymbium darker anteriorly, half the length of femur; bulb light yellow, round with sparse embolic stubble; embolus thin and dark, arising distally, almost the same length as bulb (Fig. 12A).

**Female** (paratype). General features and coloration similar to that of male (Fig. 11D–E). Measurements: total length 1.62; carapace 0.62 long, 0.70 wide; abdomen 1.00 long, 0.70 wide. Leg measurements: I 5.39 (1.40, 0.25, 1.56, 1.40, 0.78), II 5.50 (1.00, 0.25, 1.25, 1.13, 1.12, 0.75), III 3.72 (0.87, 0.25, 1.00, 1.00, 0.60), IV 5.42 (1.37, 0.25, 1.60, 1.40, 0.80). Endogyne: a pair of short, twisted, and paired coiled spermathecae, ratio of spermathecae interdistance and spermatheca width 1 : 5 (Fig. 11A).

##### Distribution.

Known only from the type locality (Fig. 29).

#### Thaiderces zuichun

Taxon classificationAnimaliaAraneaePsilodercidae

Li & Chang sp. nov.

DBC30567FEE85852825AC846CFAE94FB

http://zoobank.org/3D32A377-A33F-47B7-9844-1A286D5A7F7B

**Figure 13.**
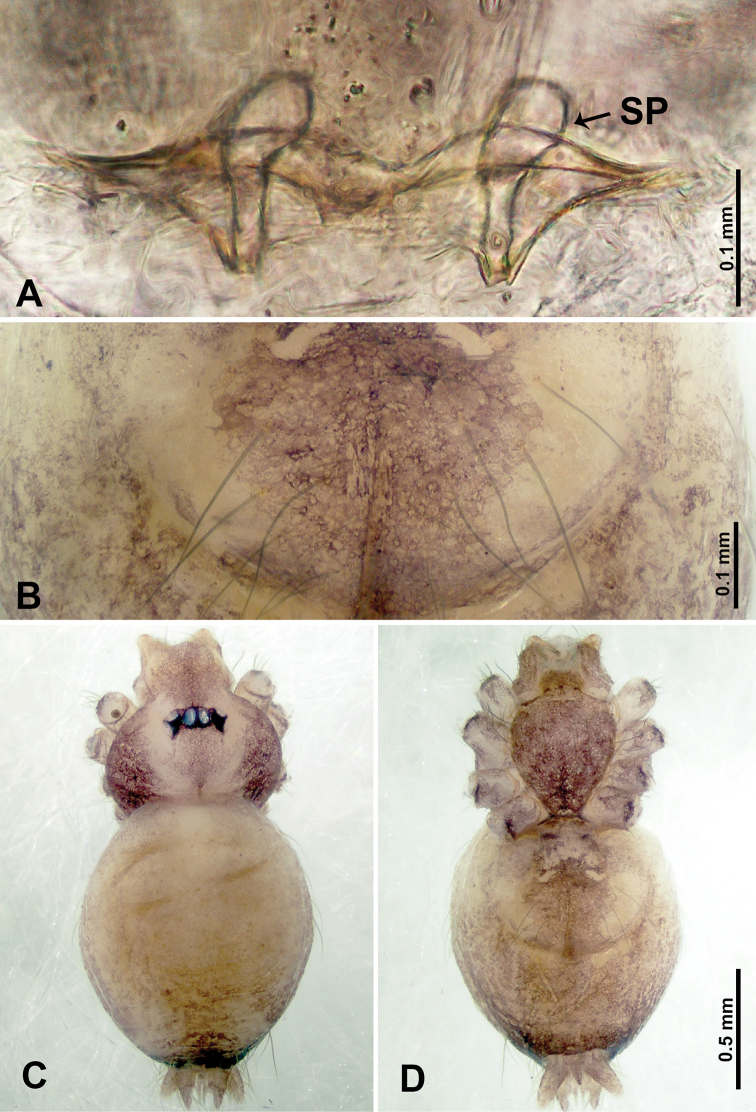
*Thaiderces zuichun* sp. nov., female paratype **A** endogyne, dorsal view **B** epigastric area, ventral view **C** habitus, dorsal view **D** habitus, ventral view. Abbreviation: SP, spermatheca.

Figs 2H , 13 , 27G , 29

##### Types.

**Holotype**: ♀ (IZCAS), Thailand, Satun Province, Thung Wa District, Cave without name, 07°3.04278'N, 99°48.03915'E, 28 m, 01.XII.2013, F. Ballarin.

##### Etymology.

The species name is a noun in apposition derived from the Chinese pinyin *zuǐchún* (lip) and refers to the overall structure of the spermathecae which is similar to a human lip.

##### Diagnosis.

*Thaiderces zuichun* sp. nov. is similar to *T. miantiao* sp. nov. but can be easily distinguished by a pair of stalked spermathecae with an oblong distal part connected to a wavy horizontal duct (vs. two pairs of strongly twisted spermathecae in *T. miantiao* sp. nov.).

##### Description.

Female. Total length 1.58; carapace 0.50 long, 0.63 wide; abdomen 1.08 long, 0.78 wide. Carapace round and brown, with three longitudinal purplish bands, median band rather pale and only half the length of the carapace, lateral bands three times wider than the median band (Fig. 13C). Chelicerae brown (Fig. 27G). Clypeus purple. Endites purple. Labium brown. Sternum with purplish pattern. Abdomen elongated, posterior with indistinct dark brown pattern (Fig. 13C), ventrum with indistinct brown pattern, anterior epigastric area forming a semi-circle (Fig. 13D). Legs uniformly brown; measurements: I missing, II 4.13 (1.09, 0.15, 1.25, 1.09, 0.55), III 3.45 (0.94, 0.16, 0.94, 0.94, 0.47), IV 4.70 (1.20, 0.20, 1.40, 1.30, 0.60). Endogyne: a pair of stalked spermathecae, with oblong distal parts, stalk almost equal in length to and half the width of the oblong distal part, connected by a wavy horizontal duct (Fig. 13A)

**Male**. Unknown.

##### Distribution.

Known only from the type locality (Fig. 29).

#### Thaiderces miantiao

Taxon classificationAnimaliaAraneaePsilodercidae

Li & Chang sp. nov.

042B2C0AE1A351F988BD56AC50B27021

http://zoobank.org/04C6EF20-9E2D-4C42-BAE2-EE2EFB19704D

**Figure 14.**
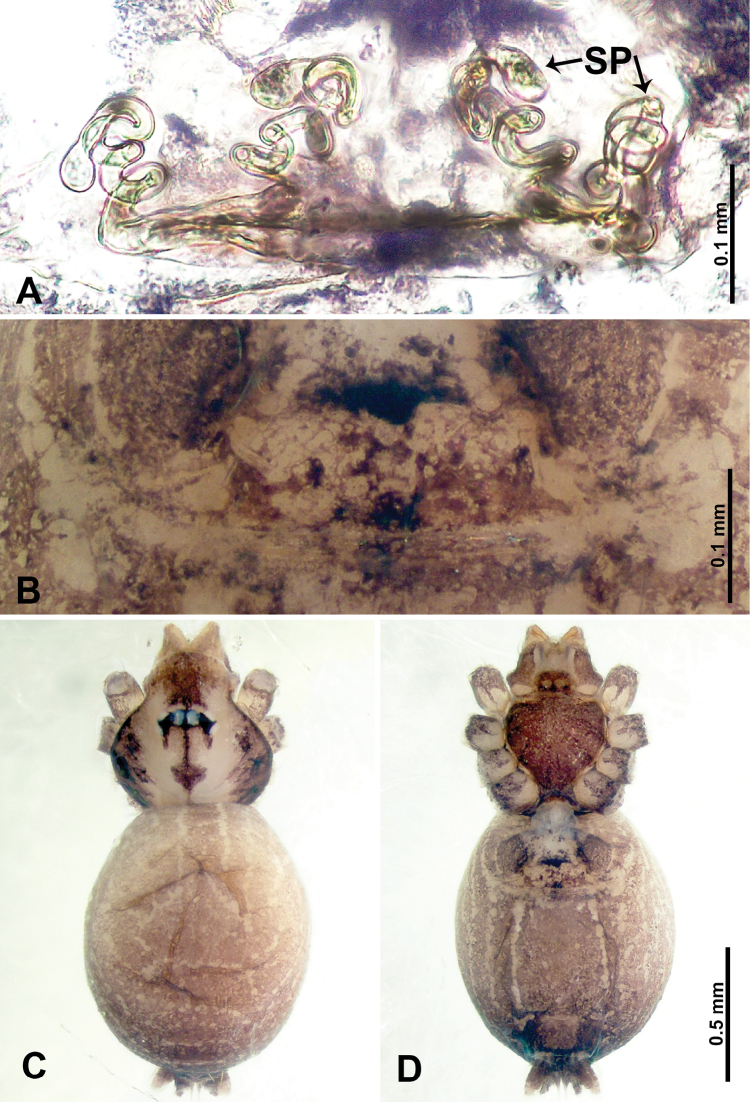
*Thaiderces miantiao* sp. nov., female paratype **A** endogyne, dorsal view **B** epigastric area, ventral view **C** habitus, dorsal view **D** habitus, ventral view. Abbreviation: SP, spermatheca.

Figs 2N , 14 , 27H , 29

##### Types.

**Holotype**: ♀ (IZCAS), Thailand, Nakhon Srithammarat Province, Thung Song District, outside of Ta Lod Cave, 08°2.3667'N, 99°44.8333'E, 120 m, 14.X.2015, Zhao Q., Zhou G., Chen Z.

##### Etymology.

The species name is a noun in apposition derived from the Chinese pinyin *miàntiáo* (noodle) and refers to the spermathecae structure which resembles curly noodles (twisted structure).

##### Diagnosis.

See diagnosis for *T. zuichun* sp. nov.

##### Description.

Female. Total length 1.56; carapace 0.54 long, 0.60 wide; abdomen 1.02 long, 0.86 wide. Carapace round and brown, with two longitudinal dark brown bands laterally, and a central dark brown patch (Fig. 14C). Chelicerae brown (Fig. 27H). Clypeus brown. Endites brown. Labium dark brown. Sternum with dark brown pattern. Abdomen elongated, with indistinct brown pattern (Fig. 14C), ventrum with two circular patches followed by an ovate epigastric area anteriorly, posterior with yellow dotted lines laterally and indistinct brown pattern (Fig. 14D). Legs uniformly brown; measurements: I 5.05 (1.20, 0.15, 1.50, 1.50, 0.70), II‒IV missing. Endogyne: two pairs of strongly twisted helical spermathecae, with globose distal part nearly the same width as the stalk, stalk en times longer than the globose head, ratio of lateral pairs of spermathecae interdistance to median pair interdistance 1 : 3.5 (Fig. 14A).

**Male**. Unknown.

##### Distribution.

Known only from the type locality (Fig. 29).

#### Thaiderces jiazi

Taxon classificationAnimaliaAraneaePsilodercidae

Li & Chang sp. nov.

855443FA9F8B540F896C345DC9E92BAE

http://zoobank.org/C21D07D6-0B6C-4AE3-847E-70B9953F5DEB

**Figure 15.**
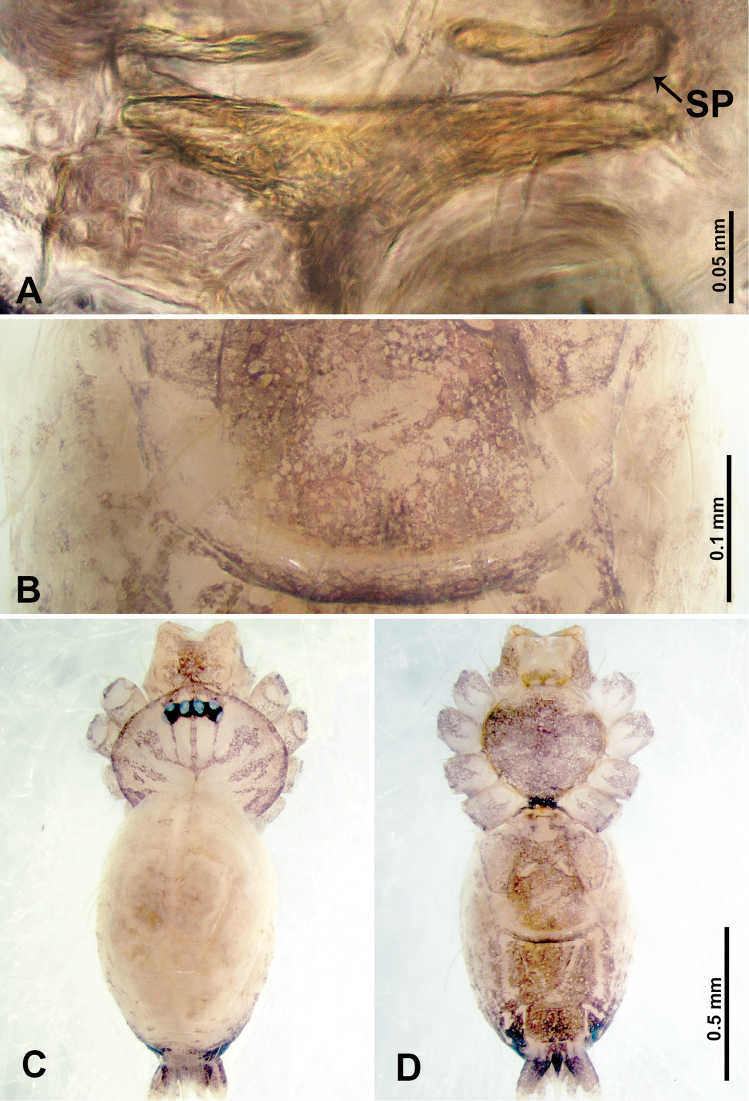
*Thaiderces jiazi* sp. nov., female paratype **A** endogyne, dorsal view **B** epigastric area, ventral view **C** habitus, dorsal view **D** habitus, ventral view. Abbreviation: SP, spermatheca.

Figs 2G , 15 , 27I , 29

##### Types.

**Holotype**: ♀ (IZCAS), Thailand, Satun Province, Khuang Kalong District, Khao Wang Cave, 06°56.3167'N, 100°1.3083'E, 127 m, 17.X.2015, Zhao Q., Zhou G., Chen Z.

##### Etymology.

The species name is a noun in apposition derived from the Chinese pinyin *jiázǐ* (clamp) and refers to the spermathecae structure which resembles a face clamp (Fig. 15A).

##### Diagnosis.

*Thaiderces jiazi* sp. nov. can be distinguished from other species by the unique pattern on the carapace, with trident purplish stripes medially and purplish stripes laterally (Fig. 15C), a pair of spermathecae that resemble a face clamp (curved perpendicularly and oppositely directed), attached to a funnel-shaped structure posteriorly (Fig. 15A) (vs. stalked spermathecae with oblong heads connected by a horizontal arched duct in *T. zuichun* sp. nov.).

##### Description.

Female. Total length 1.47; carapace 0.54 long, 0.62 wide; abdomen 0.93 long, 0.65 wide. Carapace round and pale yellow, with trident purplish stripes medially and purplish stripes laterally. Chelicerae brown (Fig. 27I). Clypeus brown medially. Endites purple. Labium yellow. Sternum with purplish pattern. Abdomen elongated, pale yellow, almost plain without distinct pattern (Fig. 15C), ventrum with purplish and yellowish indistinct pattern, with medial epigastric area semi-circular (Fig. 15D). Legs uniformly brown; measurements: I missing, II 5.27 (1.40, 0.25, 1.50, 1.37, 0.75), III 4.23 (1.12, 0.20, 1.20, 1.09, 0.62), IV 4.12 (1.09, 0.20, 1.20, 1.09, 0.54). Endogyne: a pair of stalked spermathecae curved perpendicularly and oppositely directed, stalk length is eight times the width of the spermathecae, spermathecae connected by stalks to funnel-shaped base, ratio of the width of funnel to the width of a stalked spermatheca 1 : 10 (Fig. 15A).

**Male**. Unknown.

##### Distribution.

Known only from the type locality (Fig. 29).

#### Thaiderces tuoyuan

Taxon classificationAnimaliaAraneaePsilodercidae

Li & Chang sp. nov.

438BED81919450789E0194FCC3EAFCEC

http://zoobank.org/66DE60D1-CCC5-47AE-91A2-09498FDBA3AB

**Figure 16.**
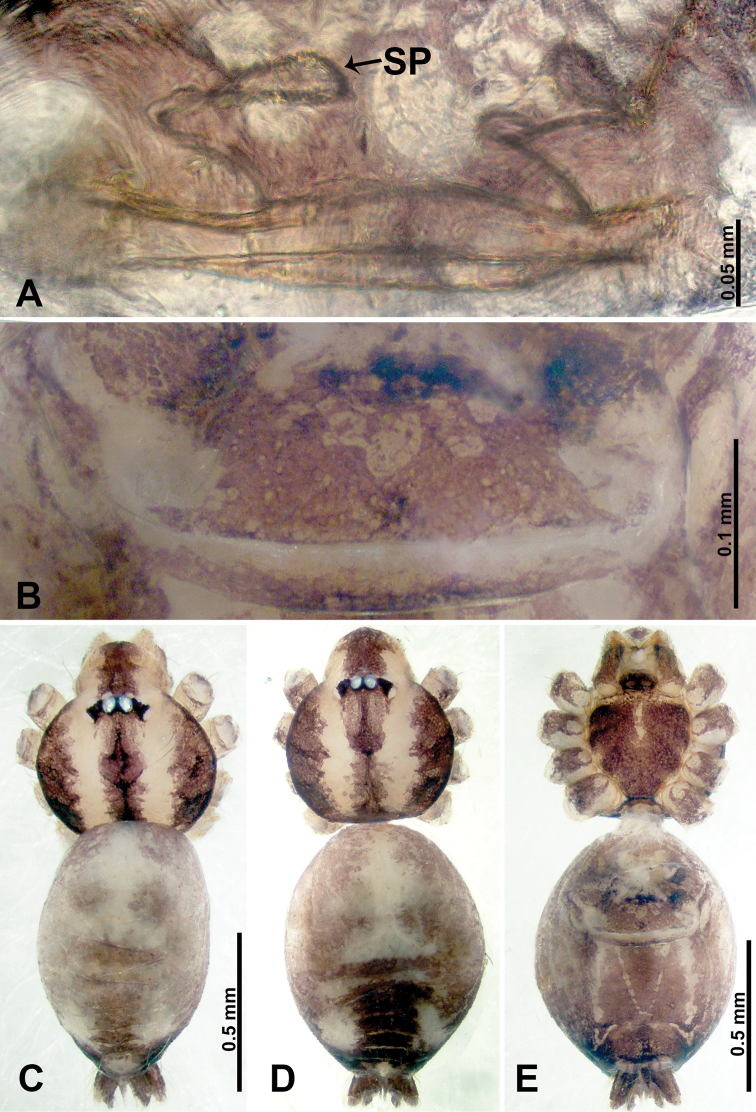
*Thaiderces tuoyuan* sp. nov., male holotype and female paratype **A** endogyne, dorsal view **B** female epigastric area, ventral view **C** male habitus, dorsal view **D** female habitus, dorsal view **E** female habitus, ventral view. Abbreviation: SP, spermatheca.

**Figure 17.**
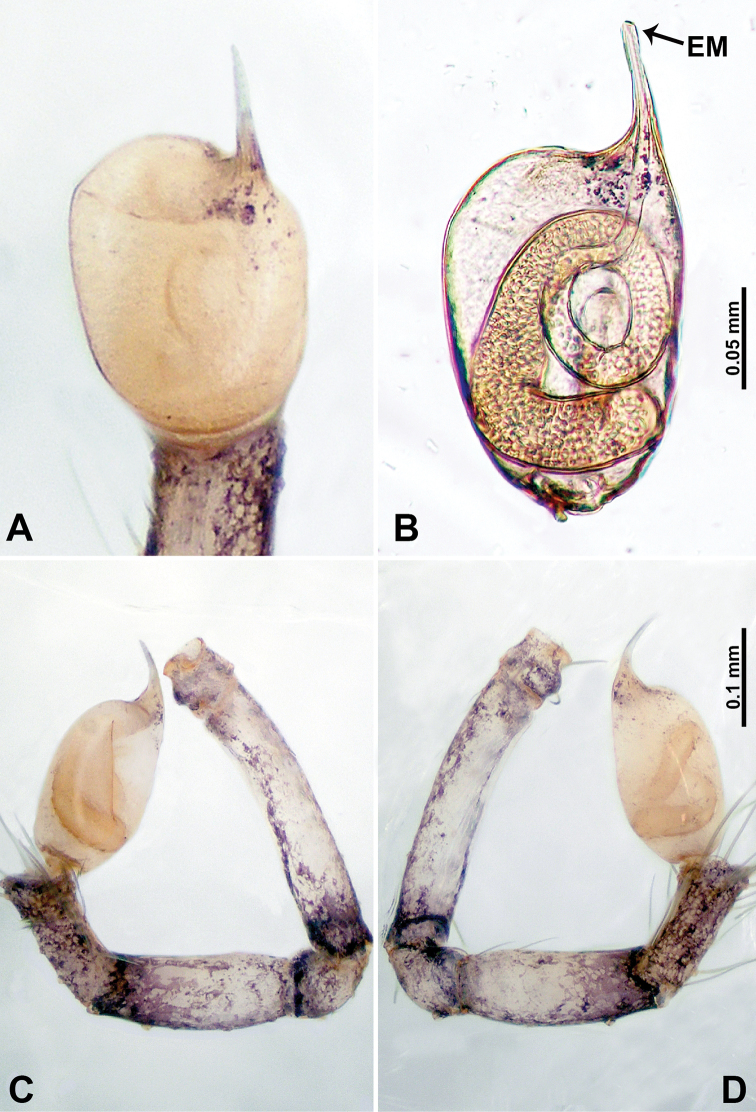
*Thaiderces tuoyuan* sp. nov., male holotype **A** palp, ventral view **B** palpal bulb, ventral view **C** palp, prolateral view **D** palp, retrolateral view. Abbreviation: EM, embolus.

Figs 1D , 2F , 16 , 17 , 28B , 29

##### Types.

**Holotype**: ♂ (IZCAS), Thailand, Yala Province, Than To District, outside Krasaeng Cave, 02°11.9998'N, 101°11.5512'E, 86 m, 25.X.2015, Yao Z. **Paratype**: 1♀ (IZCAS), same data as holotype.

##### Etymology.

The species name is a noun in apposition derived from the Chinese pinyin *tuŏyuán* (oval) and refers to the ovoid shape of the bulb.

##### Diagnosis.

*Thaiderces tuoyuan* sp. nov. is similar to *T. jian*, but males can be distinguished by the obovate bulb (vs. oblong bulb in *T. jian*), the position of the entire bulb is a mirror image of that of *T. jian* but theembolus arises from the opposite position in the two species; females can be distinguished by having one pair of spermathecae (vs. two pairs in *T. jian*).

##### Description.

**Male** (holotype). Total length 1.40; carapace 0.54 long, 0.55 wide; abdomen 0.86 long, 0.54 wide. Carapace round and brown, with three longitudinal dark brown bands of nearly equal width (Fig. 16C). Chelicerae brown (Fig. 28B). Clypeus dark brown medially and light brown laterally. Endites pale yellow. Labium dark brown. Sternum dark brown, with median pale yellow band delimitating the two halves of the sternum. Abdomen elongated, dorsum with light brown stripes posteriorly (Fig. 16C), anteroventrally with a brown semi-circle, posterior part with pair of pale yellow vertical dotted lines laterally and a V-shaped mark medially. Legs uniformly brown; measurements: I‒III missing, IV 5.38 (1.38, 0.20, 1.60, 1.40, 0.80). Palp (Fig. 17A–D): femur four times longer than patella; patella not swollen, tibia 2/3 the length of femur; cymbium scattered with concentrated purplish spots, half the length of femur; bulb light yellow, obovate with embolus arising distally; embolus short and curved, 1/3 the length of the bulb (Fig. 17C, D).

**Female** (Paratype). General features and coloration similar to that of male (Fig. 16D, E). Measurements: total length 1.62; carapace 0.50 long, 0.62 wide; abdomen 1.12 long, 0.70 wide. Leg measurements: I 4.30 (1.09, 0.20, 1.30, 1.09, 0.62), II missing, III 3.46 (0.80, 0.16, 0.94, 0.93, 0.63), IV 5.47 (1.25, 0.16, 1.56, 1.41, 1.09). Endogyne: a pair of twisted spermathecae, opposing one another, blunt distal parts 3 times wider than the width of stalks, stalk 2 times longer than the head (Fig. 16A).

##### Distribution.

Known only from the type locality (Fig. 29).

#### Thaiderces fengniao

Taxon classificationAnimaliaAraneaePsilodercidae

Li & Chang sp. nov.

B5DA30846A595FC3BBA276E6E0CF8101

http://zoobank.org/79BB2169-2674-4888-BF44-B77A406644F5

**Figure 18.**
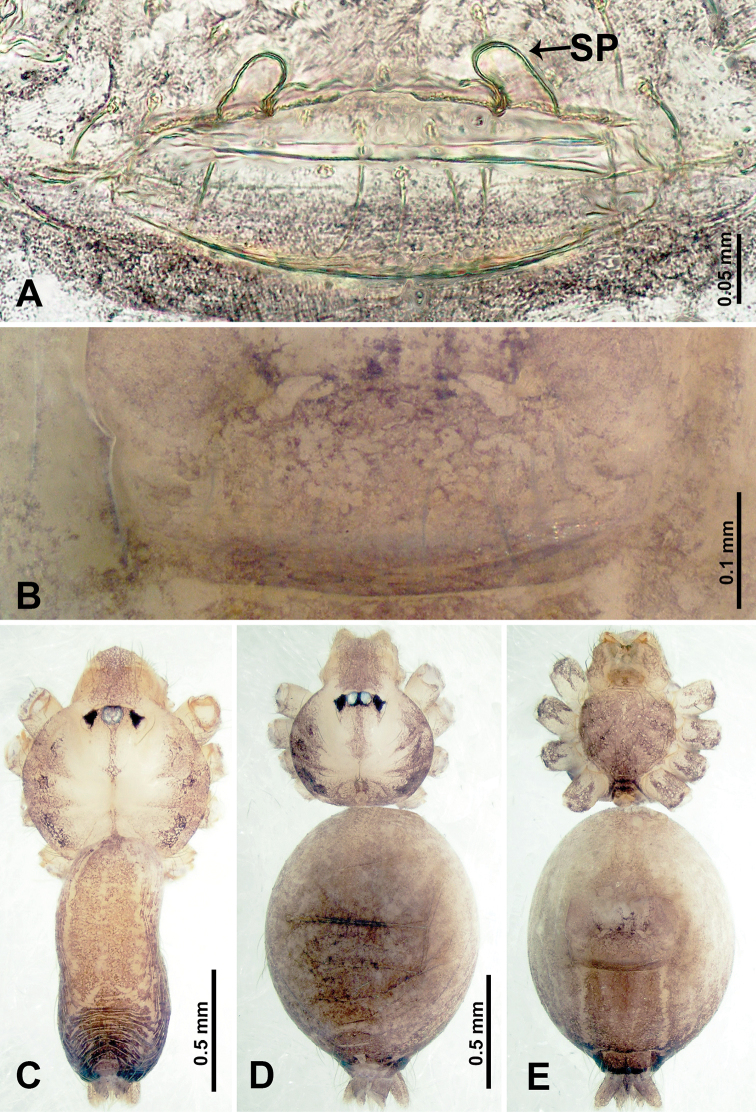
*Thaiderces fengniao* sp. nov., male holotype and female paratype **A** endogyne, dorsal view **B** female epigastric area, ventral view **C** male habitus, dorsal view **D** female habitus, dorsal view **E** female habitus, ventral view. Abbreviation: SP, spermatheca.

**Figure 19.**
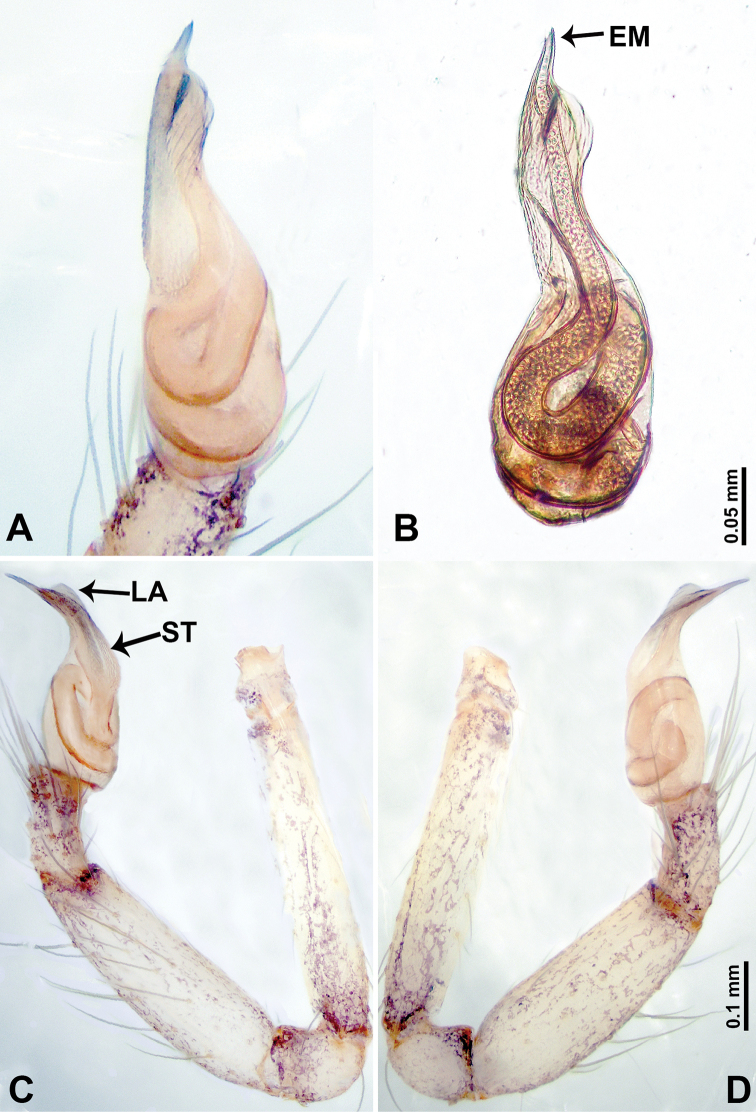
*Thaiderces fengniao* sp. nov., male holotype **A** palp, ventral view **B** palpal bulb, ventral view **C** palp, prolateral view **D** palp, retrolateral view. Abbreviations: EM, embolus, LA, laminal apophysis, ST, stubble.

Figs 1F , 2D , 18 , 19 , 27D , 29

##### Types.

**Holotype**: ♂ (IZCAS), Thailand, Kanchanaburi Province, Sai Yok District, Wang Krachae Subdistrict, unnamed Cave, 14°12.127'N, 99°01.195'E, 438 m, 1.XI.2014, Zhao H., Li Y., Chen Z. **Paratype**: 1♀ (IZCAS), same data as holotype.

##### Etymology.

The species name is a noun in apposition derived from the Chinese pinyin *fēngniăo* (hummingbird) and refers to the entire structure of the bulb, including the embolic stalk and laminar apophysis, resembling the head of a hummingbird.

##### Diagnosis.

*Thaiderces fengniao* sp. nov. is similar to *T. haima* sp. nov. but can be distinguished by a lighter color of pale yellow and purplish pattern as a whole (vs. rather darker color of brown pattern as a whole in *T. haima* sp. nov.), the embolic stubble is divided into two rows (Fig. 19C) (vs. the embolic stubble is undivided in *T. haima* sp. nov.), presence of laminar apophysis adjacent to embolus (Fig. 19C) (vs. the absence of laminar apophysis in *T. haima* sp. nov.), and the tibia is 2/3 the length of femur (vs. tibia almost equal to the length of femur in *T. haima* sp. nov.); females can be distinguished by a pair of short tubular spermathecae (vs. a pair of circular doublet spermathecae in *T. haima* sp. nov.).

##### Description.

**Male** (holotype). Total length 1.70; carapace 0.80 long, 0.78 wide; abdomen 0.90 long, 0.50 wide. Carapace round and brown, with 3 longitudinal dark brown bands, lateral bands four times wider than the median band (Fig. 18C). Chelicerae brown (Fig. 27D). Clypeus purplish medially and light brown laterally. Endites pale yellow. Labium brown. Sternum with purplish pattern. Abdomen elongated, dorsum with indistinct purplish pattern, posterior with dark purple stripes, ventrum with indistinct brown pattern. Legs uniformly brown; measurements: I 7.18 (1.87, 0.25, 2.18, 1.88, 1.00), II missing, III 5.72 (1.60, 0.25, 1.62, 1.50, 0.75), IV 9.63 (2.60, 0.31, 2.81, 2.66, 1.25). Palp (Fig. 19A–D): femur four times longer than patella; patella not swollen, tibia 2/3 the length of femur; cymbium lightly scattered with purplish spots, 1/3 the length of the femur; bulb light yellow, lanceolate with bent distal part, presence of two rows of embolic stubble on distal part of embolic stalk, embolic stalk half the width of bulb, embolus short, arising distally, with laminar apophysis adjacent to embolus, greatly resembles the head of hummingbird with the distinct beak (Fig. 19C, D).

**Female** (Paratype). General features and coloration similar to that of male (Fig. 18D–E). Measurements: total length 1.85; carapace 0.75 long, 0.60 wide; abdomen 1.10 long, 1.00 wide. Leg measurements: I 6.80 (1.80, 0.25, 2.00, 1.75, 1.00), II 5.26 (1.40, 0.20, 1.56, 1.30, 0.80), III 4.35 (1.28, 0.20, 1.12, 1.13, 0.62), IV 6.18 (1.56, 0.20, 1.80, 1.62, 1.00). Endogyne: a pair of short, tubular spermathecae bent towards each other, with a length 2 times the width, ratio of interdistance between spermatheca and the width of spermatheca 1 : 3.75 (Fig. 18A).

##### Distribution.

Known only from the type locality (Fig. 29).

#### Thaiderces haima

Taxon classificationAnimaliaAraneaePsilodercidae

Li & Chang sp. nov.

E9FF27B6591E56A4A3B50D0DAB4D2E84

http://zoobank.org/91D1E11C-CF9D-48FB-86D0-7720F2ACA4B8

**Figure 20.**
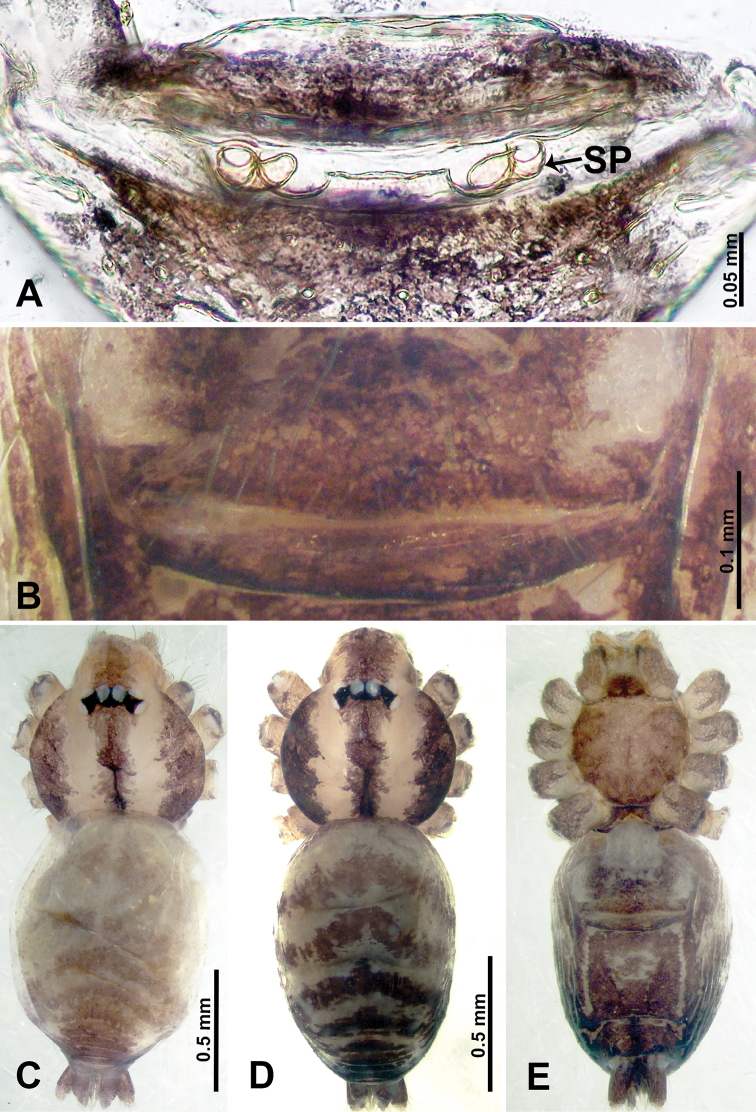
*Thaiderces haima* sp. nov., male holotype and female paratype **A** endogyne, dorsal view **B** female epigastric area, ventral view **C** male habitus, dorsal view **D** female habitus, dorsal view **E** female habitus, ventral view. Abbreviation: SP, spermatheca.

**Figure 21.**
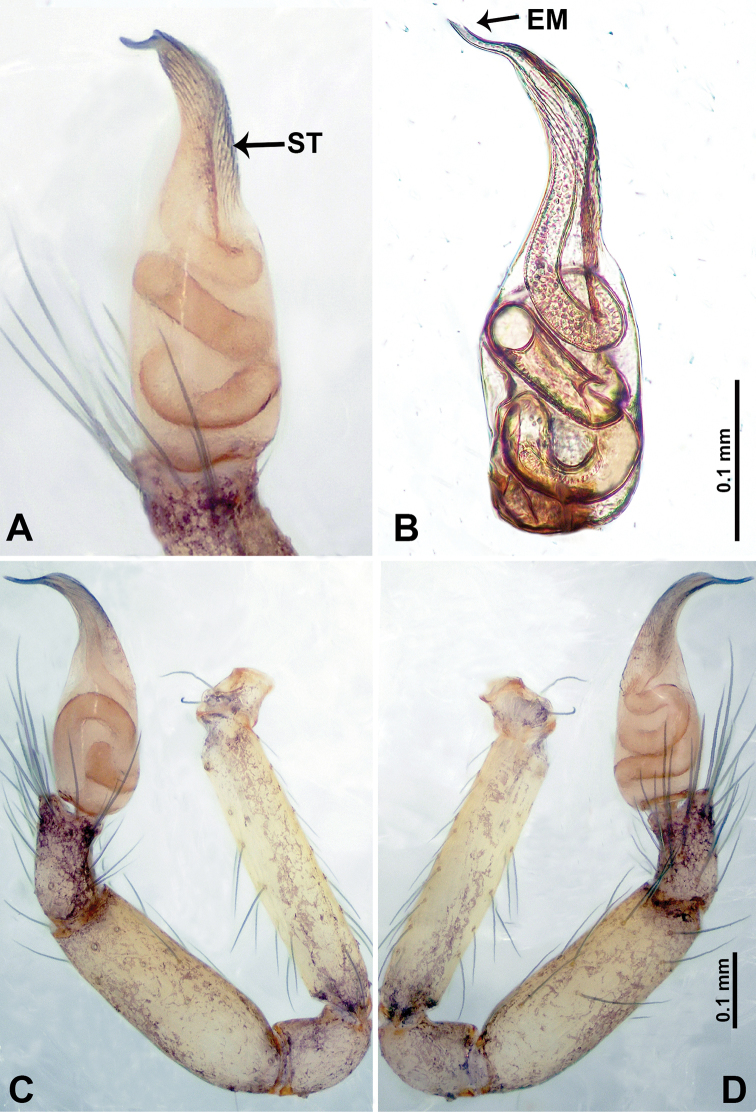
*Thaiderces haima* sp. nov., male holotype **A** palp, ventral view **B** palpal bulb, ventral view **C** palp, prolateral view **D** palp, retrolateral view. Abbreviations: EM, embolus, ST, stubble.

Figs 1L , 2I , 20 , 21 , 28C , 29

##### Types.

**Holotype**: ♂ (IZCAS), Thailand, Tak Province, Uaphang District, Umphang Subdistrict, Huai Lao Poo Cave, 15°57.680'N, 98°52.510'E, 534 m, 16.XI.2016, Zhao H., Li Y., Chen Z. **Paratype**: 1♀ (IZCAS), same data as holotype.

##### Etymology.

The species name is a noun in apposition derived from the Chinese pinyin hăIimă (seahorse) and refers to the distal bending of bulb that resembles the head of a seahorse.

##### Diagnosis.

See diagnosis of *T. fengniao* sp. nov.

##### Description.

**Male** (holotype). Total length 1.80; carapace 0.60 long, 0.70 wide; abdomen 1.20 long, 0.90 wide. Carapace round and brown, with three longitudinal dark brown bands, lateral bands two times wider than the median band (Fig. 20C). Chelicerae brown (Fig. 28C). Clypeus dark brown medially and light brown laterally. Endites light brown. Labium dark brown. Sternum with dark brown pattern. Abdomen elongated, dorsum with indistinct brown pattern, posterior with brown stripes, anteroventrally with dark brown semi-circle, posterior part with pair of pale yellow vertical dotted lines laterally and a funnel-shaped mark medially. Legs uniformly brown; measurements: I missing, II missing, III 4.78 (1.28, 0.25, 1.38, 1.25, 0.62), IV 7.12 (1.75, 0.31, 2.12, 2.00, 0.94). Palp (Fig. 21A–D): femur 3 times longer than patella; patella not swollen, tibia almost equal in length to femur; cymbium scattered with purplish spots anteriorly, 1/4 the length of femur; bulb brown, lanceolate with bent distal part, presence of embolic stubble on embolic stalk, embolic stalk 1/3 the width of the bulb, embolus short, arises distally, resembles the head of a seahorse (Fig. 21A).

**Female** (paratype). General features and coloration similar to that of the male (Fig. 20D, E). Measurements: total length 1.60; carapace 0.50 long, 0.63 wide; abdomen 1.10 long, 0.70 wide. Leg measurements: I missing, II 4.29 (1.09, 0.16, 1.25, 1.09, 0.70), III 4.92 (1.25, 0.16, 1.41, 1.30, 0.80), IV missing. Endogyne: two pairs of circular spermathecae, lateral spermathecae embedded with ovoid duct (Fig. 20A).

##### Distribution.

Known only from the type locality (Fig. 29).

#### Thaiderces chujiao

Taxon classificationAnimaliaAraneaePsilodercidae

Li & Chang sp. nov.

1ED96F2A3587523FAE36B0918105F127

http://zoobank.org/3BA4354E-FE20-4DAE-850F-6082A9443508

Figs 22 , 28A , 29

##### Types.

**Holotype**: ♀ (IZCAS), Thailand, Chiangmai Province, Mae Cham District, Jeep track, 18°31.677'N, 98°29.963'E, 1649 m, 14.X.2014, Zhao H., Li Y., Chen Z.

##### Etymology.

**Figure 22.**
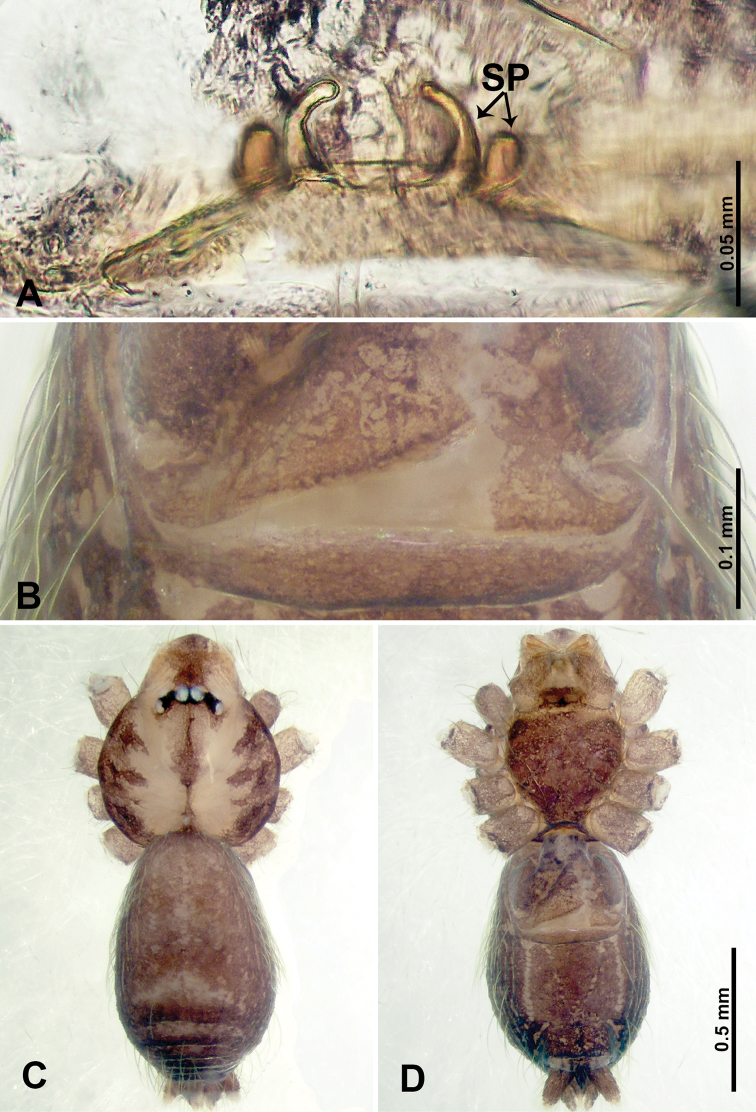
*Thaiderces chujiao* sp. nov., female paratype **A** endogyne, dorsal view **B** epigastric area, ventral view **C** habitus, dorsal view **D** habitus, ventral view. Abbreviation: SP, spermatheca.

The species name is a noun in apposition derived from the Chinese pinyin *chùjiăo* (antenna) and refers to the structure of the spermathecae that resembles two pairs of clavate antennae (Fig. 22A).

##### Diagnosis.

*Thaiderces chujiao* sp. nov. can be distinguished from other species by the two pairs of tubular spermathecae resembling two pairs of clavate antennae, lateral pair half the length of the median pair (Fig. 22A) (vs. circular spermathecae with with ovoid duct in *T. haima* sp. nov.).

##### Description.

**Female** (holotype). Total length 1.62; carapace 0.60 long, 0.70 wide; abdomen 1.02 long, 0.60 wide. Carapace round and brown, with three longitudinal dark brown bands, lateral bands three times wider than the median band. Chelicerae brown (Fig. 28A). Clypeus dark brown medially and light brown laterally. Endites brown. Labium dark brown. Sternum with dark brown pattern. Abdomen elongated, with dark brown pattern (Fig. 22C), anteroventrally with semi-circular light brown epigastric area medially, posterior part with pair of light brown vertical dotted lines laterally and a V-shaped mark medially (Fig. 22D). Legs uniformly brown; measurements: I 4.84 (1.25, 0.31, 1.41, 1.09, 0.78), II 3.87 (1.00. 0.20, 1.10. 0.94, 0.63), III 3.31 (0.88, 0.25, 0.88, 0.80, 0.50), IV 4.46 (1.09, 0.25, 1.25, 1.12, 0.75). Endogyne: two pairs of tubular spermathecae, lateral pair half the length of the median pair, median pair curved and opposing one another, half the width of lateral pair (Fig. 22A).

**Male**. Unknown.

##### Distribution.

Known only from the type locality (Fig. 29).

#### Thaiderces thamphadaengensis

Taxon classificationAnimaliaAraneaePsilodercidae

Li & Chang sp. nov.

F10E295FEC0B5499A15626827AF08ED1

http://zoobank.org/30F678E7-EC6E-4B95-8418-4CDCCF8730A8

**Figure 23.**
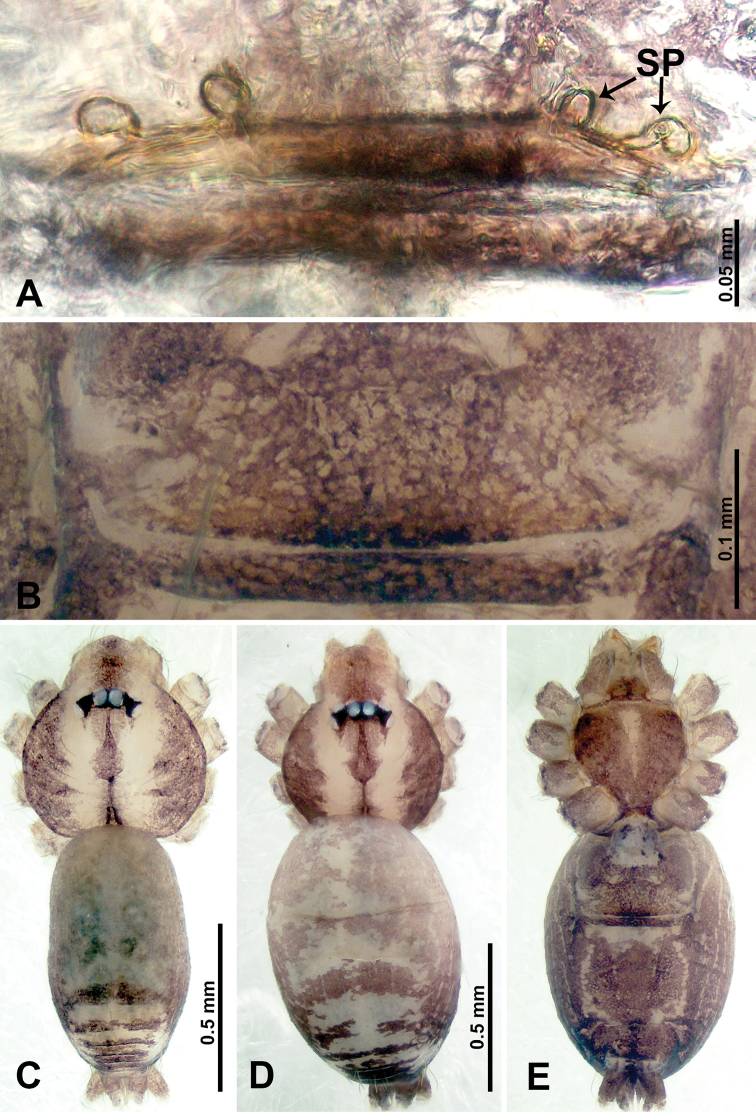
*Thaiderces thamphadaengensis* sp. nov., male holotype and female paratype **A** endogyne, dorsal view **B** female epigastric area, ventral view **C** male habitus, dorsal view **D** female habitus, dorsal view **E** female habitus, ventral view. Abbreviation: SP, spermatheca.

**Figure 24.**
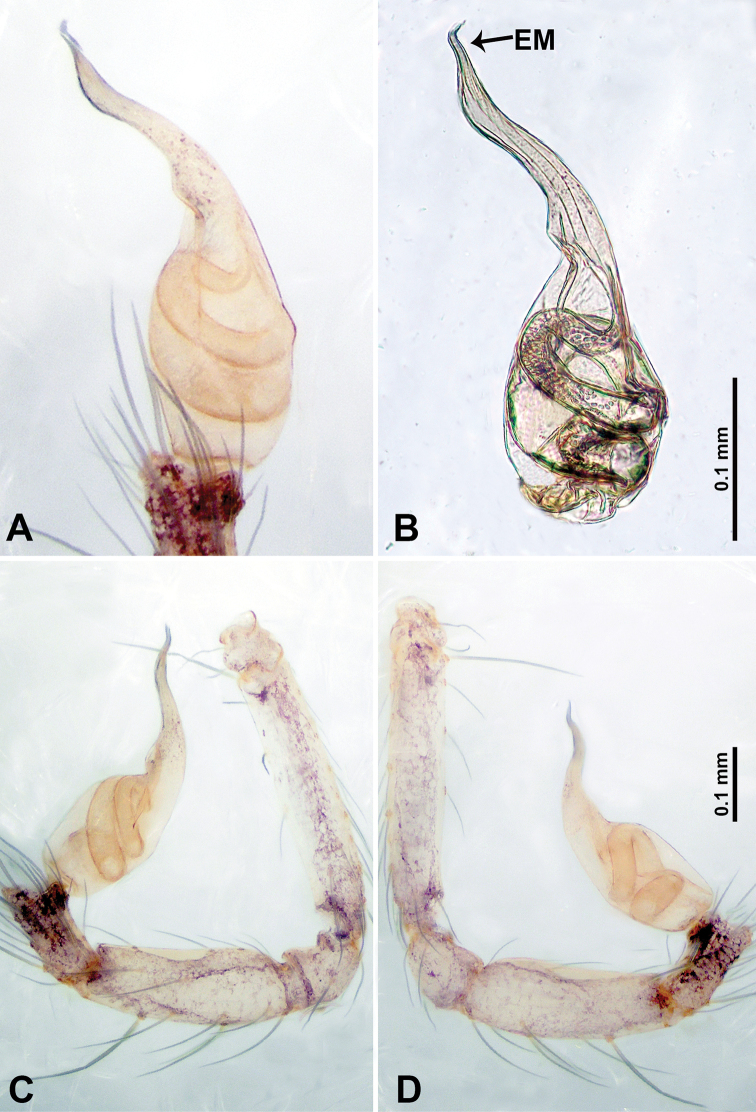
*Thaiderces thamphadaengensis* sp. nov., male holotype **A** palp, ventral view **B** palpal bulb, ventral view **C** palp, prolateral view **D** palp, retrolateral view. Abbreviation: EM, embolus.

Figs 1I , 2K , 23 , 24 , 28D , 29

##### Types.

**Holotype**: ♂ (IZCAS), Thailand, Mae Hong Muang Province, Muang District, Mok Jumpae Subdistrict, Tham PhaDaeng Cave, 19°25.395'N, 97°59.057'E, 293 m, 21.XI.2016, Zhao H., Li Y., Chen Z. **Paratype**: 1♀ (IZCAS), same data as holotype.

##### Etymology.

The species name is an adjective referring to the type locality.

##### Diagnosis.

*Thaiderces thamphadaengensis* sp. nov. can be distinguished from other species of the genus by the distinct long, flat and tapered embolic stalk (vs. embolic stalk not flat and tapered in other congeners); females can be distinguished by two pairs of spermathecae, lateral pair with short stalks, median pair circular (vs. one pair of similar spermathecae, tubular or twisted spermathecae in other congeners).

##### Description.

**Male** (holotype). Total length 1.30; carapace 0.60 long, 0.59 wide; abdomen 0.70 long, 0.44 wide. Carapace round and brown, with three longitudinal dark brown bands, lateral bands three times wider than the median band (Fig. 23C). Chelicerae brown (Fig. 28D). Clypeus dark brown medially and light brown laterally. Endites brown. Labium dark brown. Sternum with dark brown pattern, delimiting an inverted triangle medially. Abdomen elongated, dorsum with indistinct brown pattern, posterior with brown stripes, anteroventrally with dark brown semi-circle, posterior with pair of pale yellow vertical dotted lines laterally. Legs uniformly brown; measurements: I missing, II 4.45 (1.25, 0.20, 1.25, 1.00, 0.75), III 3.70 (1.00, 0.20, 1.01, 0.94, 0.55), IV 5.66 (1.40, 0.25, 1.63, 1.50, 0.88). Palp (Fig. 24A–D): femur 5 times longer than patella; patella not swollen, tibia 2/3 the length of femur; cymbium with dark brown spots anteriorly, 1/3 the length of femur; bulb pale yellow, spatulate, with elongated, tapered embolic stalk, embolic stalk half the width of bulb, embolus short, arises distally from bulb (Fig. 24A).

**Female** (paratype). General features and coloration similar to that of male (Fig. 23D, E). Measurements: total length 1.60; carapace 0.50 long, 0.62 wide; abdomen 1.10 long, 0.70 wide. Leg measurements: I missing, II 3.99 (1.09, 0.25, 1.12, 0.90, 0.63), III 3.38 (0.94, 0.20, 0.87, 0.87, 0.50), IV 4.63 (1.09, 0.16, 1.38, 1.25, 0.75). Endogyne: two pairs of spermathecae, lateral pair with short stalks bearing a globose distal part, head two times longer and wider than stalk, median pair circular, ratio of the interdistance of lateral pair to interdistance of median pair 1 : 1.50 (Fig. 23A).

##### Distribution.

Known only from the type locality (Fig. 29).

#### Thaiderces thamphrikensis

Taxon classificationAnimaliaAraneaePsilodercidae

Li & Chang sp. nov.

6806751F5E3F5C999EA1DBA5823196CA

http://zoobank.org/9AA13536-78A0-4B01-911C-D493339AAB9A

**Figure 25.**
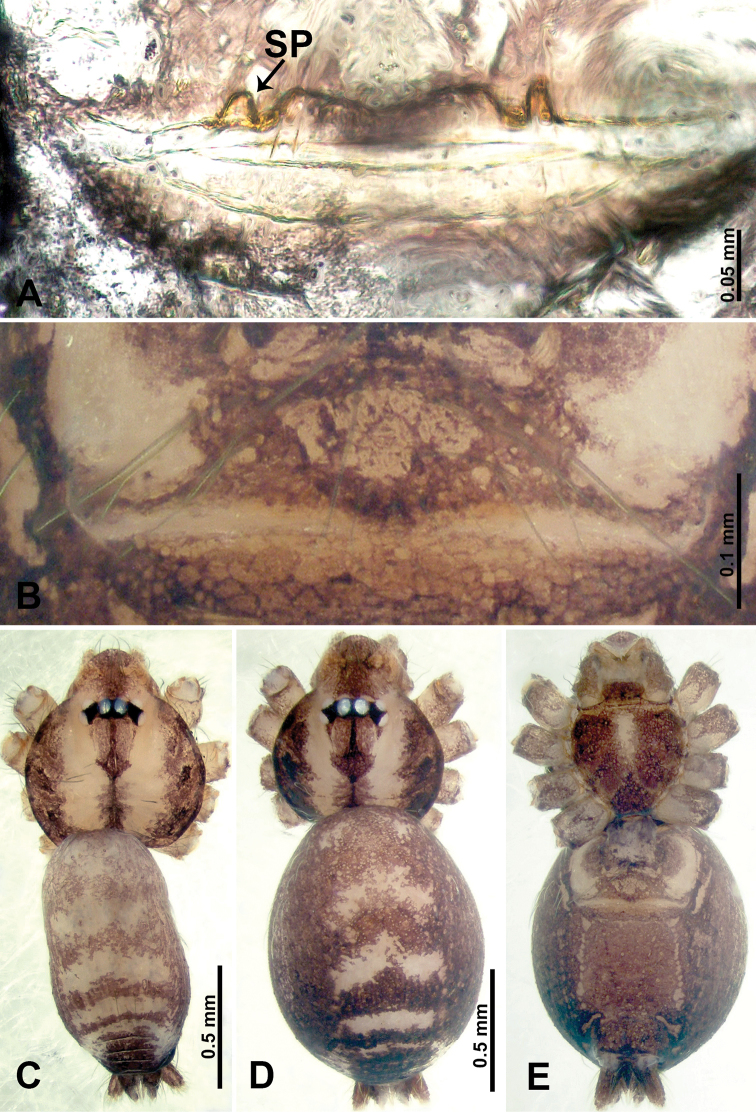
*Thaiderces thamphrikensis* sp. nov., male holotype and female paratype **A** endogyne, dorsal view **B** female epigastric area, ventral view **C** male habitus, dorsal view **D** female habitus, dorsal view **E** female habitus, ventral view. Abbreviation: SP, spermatheca.

**Figure 26.**
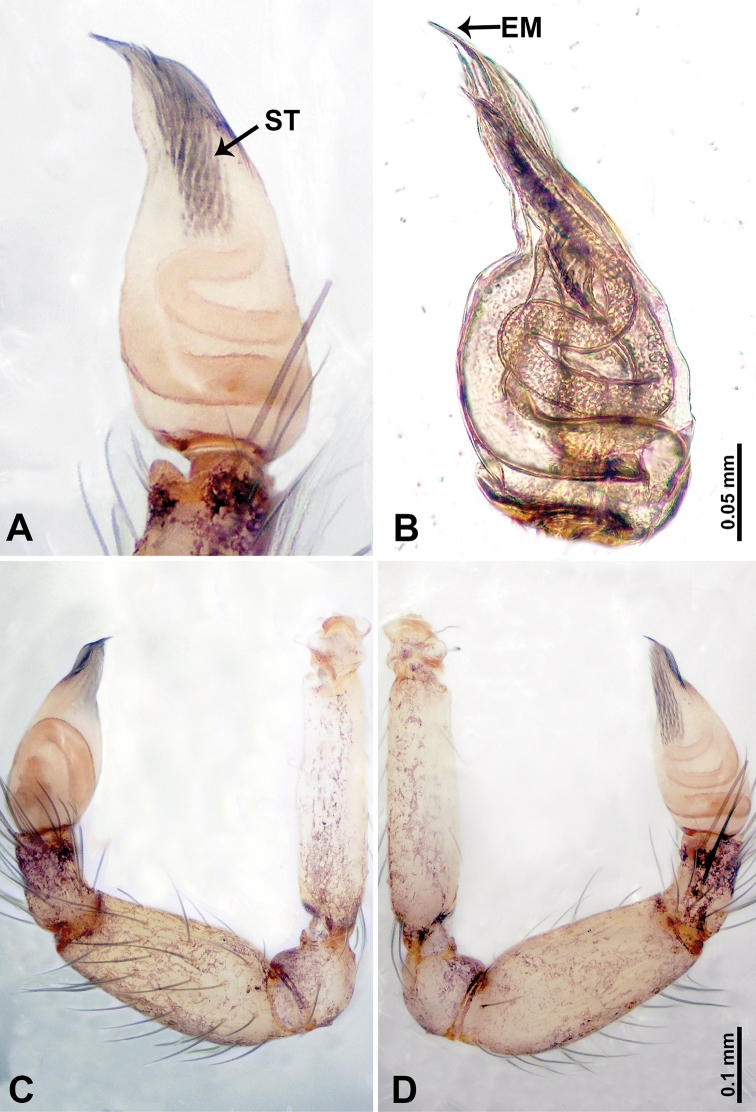
*Thaiderces thamphrikensis* sp. nov., male holotype **A** palp, ventral view **B** palpal bulb, ventral view **C** palp, prolateral view **D** palp, retrolateral view. Abbreviations: EM, embolus, ST, stubble.

**Figure 27.**
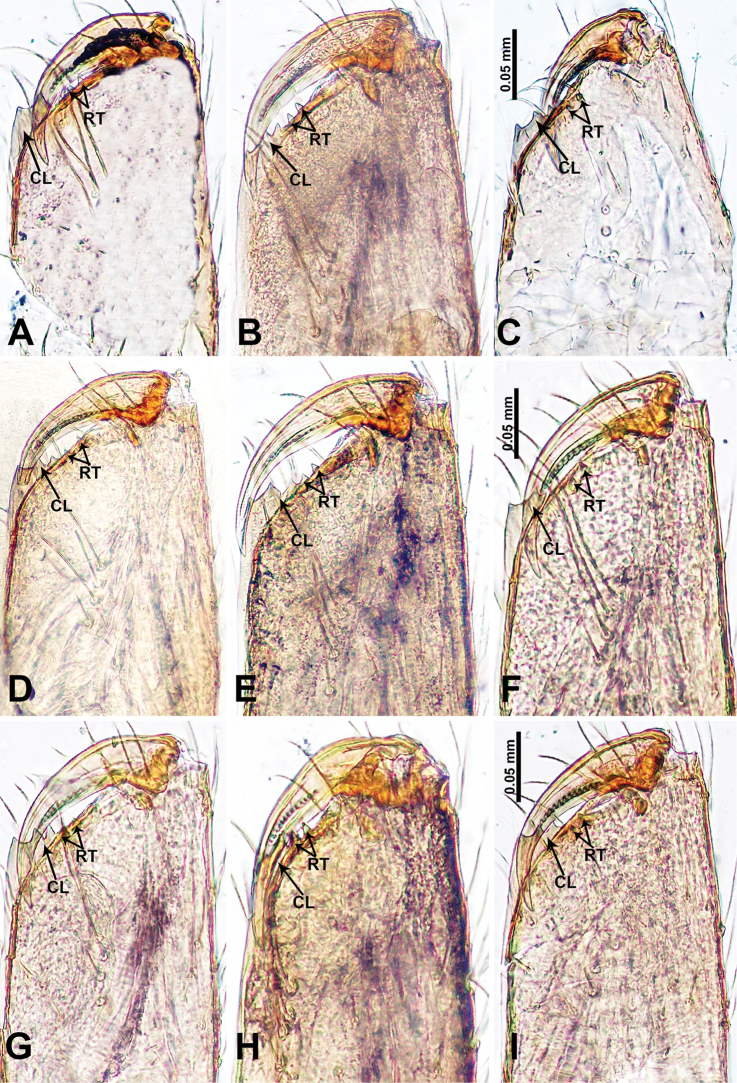
Chelicerale retromargin, posterior view **A***Thaiderces thamphrikensis* sp. nov. **B***T. yancong* sp. nov. **C***T. shuzi* sp. nov. **D***T. fengniao* sp. nov. **E***T. peterjaegeri* sp. nov. **F***T. ngalauindahensis* sp. nov. **G***T. zuichun* sp. nov. **H***T. miantiao* sp. nov. **I***T. jiazi* sp. nov. Abbreviations: CL, cheliceral lamina, RT, retromarginal teeth.

Figs 1K , 2C , 25 , 26 , 27A , 29

##### Types.

**Holotype**: ♂ (IZCAS), Thailand, Phitsanulok Province, Nakhothai District, Ban Tham Phrik Village, Tham Phrik Cave, 16°55.024'N, 100°42.173'E, 610 m, 17.X.2014, Zhao H., Li Y., Chen Z. **Paratype**: 1♀ (IZCAS), same data as holotype.

##### Etymology.

The species name is an adjective referring to the type locality.

##### Diagnosis.

*Thaiderces thamphrikensis* sp. nov. is similar to *T. vulgaris* but can be distinguished by a short embolus (embolus tip does not exceed the perimeter or bulb) (Fig. 26C) (vs. long embolus in *T. vulgaris* (embolus tip exceeds perimeter of bulb)), aligned arrangement of embolic stubble on embolic stalk (vs. embolic stubble divided into two rows in *T. vulgaris*), and palpal tibia is longer than the entire bulb (vs. palpal tibia is shorter than the entire bulb); females can be distinguished by a pair of tubular spermathecae laterally, connected with wavy horizontal ducts medially (vs. two pairs of spermathecae with lateral pair shorter than median pair).

##### Description.

**Male** (holotype). Total length 1.62; carapace 0.62 long, 0.70 wide; abdomen 1.00 long, 0.55 wide. Carapace round and brown, with three longitudinal dark brown bands, lateral bands almost equally wide with the median band (Fig. 25C). Chelicerae brown (Fig. 27A). Clypeus dark brown medially and light brown laterally. Endites brown. Labium dark brown. Sternum with dark brown pattern, delimiting an inverted triangle medially. Abdomen elongated, dorsum with dark brown striped pattern, posterior with brown stripes, anteroventrally dark brown with pair of light brown kidney-shaped marks, followed by a horizontal, linear brown pattern medially, posterior part with pair of light brown vertical dotted lines laterally. Legs uniformly brown; measurements: I‒II missing, III 3.50 (0.25, 0.25, 1.30, 1.10, 0.60), IV missing. Palp (Fig. 26A–D): femur 4 times longer than patella; patella not swollen, tibia almost equal in length to femur, swollen; cymbium scattered with dark brown spots, 1/3 the length of femur; bulb pale yellow, pyriform, with a patch of aligned embolic stubble distally; embolus short and bent, arises distally.

**Female** (paratype). General features and coloration similar to that of male (Fig. 25D, E). Measurements: total length 2.60; carapace 1.40 long, 0.62 wide; abdomen 1.20 long, 0.87 wide. Leg measurements: I 5.40 (1.40, 0.20, 1.60, 1.40, 0.80), II 4.11 (1.00, 0.20, 1.20, 1.09, 0.62), III 4.74 (2.40, 0.15, 0.94, 0.78, 0.47), IV 5.13 (1.25, 0.16, 1.56, 1.41, 0.75). Endogyne: a pair of tubular spermathecae and connected with wavy horizontal ducts medially, lateral spermathecae seven times thinner than the width of the horizontal ducts (Fig. 25A).

##### Distribution.

Known only from the type locality (Fig. 29).

#### Thaiderces rimbu

Taxon classificationAnimaliaAraneaePsilodercidae

(Deeleman-Reinhold, 1995) comb. nov.

180D9CDBAAE85476BD3A50CC46286A02

Psiloderces rimbu Deeleman-Reinhold, 1995: 25, figs 54, 55.

##### Description.

Described by Deeleman-Reinhold (1995). Diagnostic features are discussed under *T. ngalauindahensis* sp. nov.

##### Distribution.

Indonesia.

##### Remarks.

This species is transferred to *Thaiderces* due to the similarity of somatic morphology and diagnostic features of the type species of the genus.

#### Thaiderces djojosudharmoi

Taxon classificationAnimaliaAraneaePsilodercidae

(Deeleman-Reinhold, 1995) comb. nov.

22BE72CF22F453038314A9C6D09F1C84

Psiloderces djojosudharmoi Deeleman-Reinhold, 1995: 21, figs 38–42.

##### Description.

Described by Deeleman-Reinhold (1995). Diagnostic features are discussed under *T. yangcong* sp. nov.

##### Distribution.

Indonesia.

##### Remarks.

The somatic morphology and diagnostic features are similar to the type species of the genus; therefore, we transfer it to *Thaiderces*.

**Figure 28.**
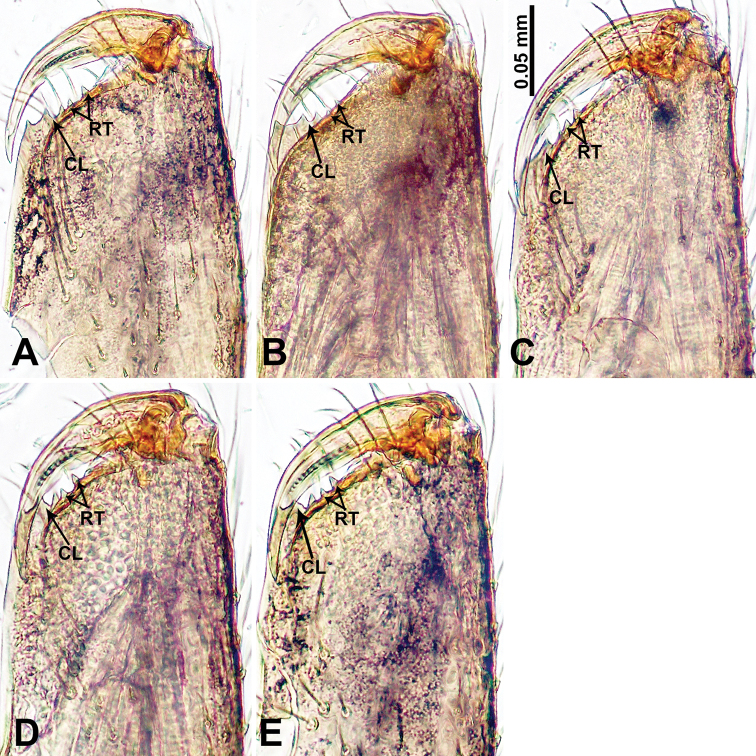
Chelicerae, posterior view **A***Thaiderces chujiao* sp. nov. **B***T. tuoyuan* sp. nov. **C***T. haima* sp. nov. **D***T. thamphadaengensis* sp. nov. **E***T. ganlan* sp. nov. Abbreviations: CL, cheliceral laminal, RT, retromargin teeth.

**Figure 29.**
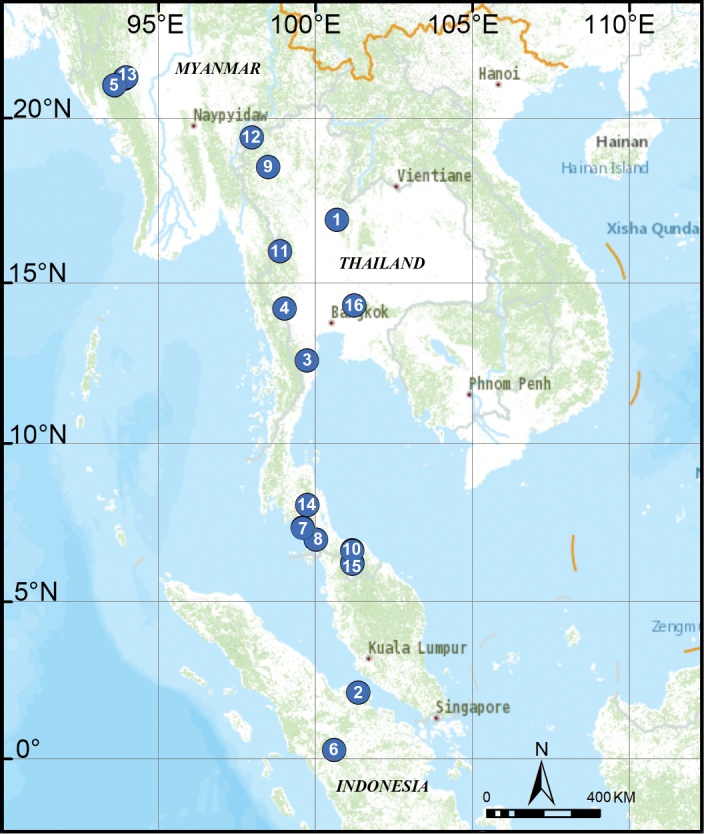
Distribution of *Thaiderces* species in Southeast Asia. **1***T. thamphrikensis* sp. nov. **2***T. yangcong* sp. nov. **3***T. shuzi* sp. nov. **4***T. fengniao* sp. nov. **5***T. peterjaegeri* sp. nov. **6***T. ngalauindahensis* sp. nov. **7***T. zuichun* sp. nov. **8***T. jiazi* sp. nov. **9***T. chujiao* sp. nov. **10***T. tuoyuan* sp. nov. **11***T. haima* sp. nov. **12***T. thamphadaengensis* sp. nov. **13***T. ganlan* sp. nov. **14***T. miantiao* sp. nov. **15***T. jian***16***T. vulgaris*. Two species not included: *T. rimbu*, *T. djojosudharmoi*.

## Supplementary Material

XML Treatment for Thaiderces

XML Treatment for Thaiderces shuzi

XML Treatment for Thaiderces peterjaegeri

XML Treatment for Thaiderces ganlan

XML Treatment for Thaiderces ngalauindahensis

XML Treatment for Thaiderces yangcong

XML Treatment for Thaiderces zuichun

XML Treatment for Thaiderces miantiao

XML Treatment for Thaiderces jiazi

XML Treatment for Thaiderces tuoyuan

XML Treatment for Thaiderces fengniao

XML Treatment for Thaiderces haima

XML Treatment for Thaiderces chujiao

XML Treatment for Thaiderces thamphadaengensis

XML Treatment for Thaiderces thamphrikensis

XML Treatment for Thaiderces rimbu

XML Treatment for Thaiderces djojosudharmoi
